# DNA-damage dependent isoform switching modulates RIF1 DNA repair complex assembly and phase separation

**DOI:** 10.1016/j.jbc.2025.110857

**Published:** 2025-10-24

**Authors:** Adenine Si-Hui Koo, Weiyan Jia, Sang Hwa Kim, Mark Scalf, Claire E. Boos, Yuhong Chen, Demin Wang, Andrew F. Voter, Aditya Bajaj, Lloyd M. Smith, James L. Keck, Christopher J. Bakkenist, Lin Guo, Randal S. Tibbetts

**Affiliations:** 1 Department of Human Oncology, School of Medicine and Public Health, University of Wisconsin-Madison, Madison, Wisconsin, USA; 2 Department of Chemistry, University of Wisconsin-Madison, Madison, Wisconsin, USA; 3 Versiti Blood Research Institute, Milwaukee, Wisconsin, USA; 4 Department of Microbiology and Immunology, Medical College of Wisconsin, Milwaukee, Wisconsin, USA; 5 Department of Biomolecular Chemistry, University of Wisconsin-Madison, Madison, Wisconsin, USA; 6 Department of Biochemistry and Molecular Biology, Thomas Jefferson University, Philadelphia, Pennsylvania, USA; 7 Department of Radiation Oncology, University of Pittsburgh, Pittsburgh, Pennsylvania, USA

**Keywords:** DNA damage, alternative splicing, RNA binding protein, cell signaling, chromatin, RIF1, MDC1

## Abstract

How RIF1 (RAP1 interacting factor 1) fulfills its diverse roles in DNA double-strand break repair, DNA replication, and nuclear organization remains elusive. Here, we show that alternative splicing of a cassette exon (Ex32) encoding a Ser/Lys-rich cassette in the RIF1 C-terminal domain (CTD) gives rise to RIF1-Long (RIF1-L) and RIF1-Short (RIF1-S) isoforms with different functional characteristics. We demonstrate that *RIF1*-Ex32 splice-in is mediated by an exonic splicing enhancer that is recognized by the serine and arginine rich splicing factor 1 (SRSF1) and antagonized by SRSF3 and SRSF7. Exposure to DNA damage inhibited Ex32 splice-in, potentiated the association of SRSF3 and SRSF7 with RIF1 pre-mRNA, and caused an increase in RIF1-S protein expression, which was also observed across a diverse set of primary cancers. Isoform-specific proteomic analyses revealed RIF1-L preferentially associated with mediator of DNA damage checkpoint 1 (MDC1) and sustained MDC1 focus formation to a greater extent than RIF1-S. We further show that the Ser/Lys-rich cassette stabilized a novel phase separation activity of the RIF1 CTD and enhanced RIF1-L chromatin retention, which was reversed by cyclin-dependent kinase 1–dependent phosphorylation of the RIF1 CTD in response to G_2_ DNA damage checkpoint inhibition. These combined findings suggest DNA damage–dependent *RIF1* alternative splicing contributes to RIF1 functional diversification in genome protection.

Originally identified as a telomere-binding factor in *Saccharomyces cerevisiae*, the RIF1 (RAP1-interacting factor 1) gene encodes a ∼270 kDa protein that fulfills diverse roles in eukaryotic genome maintenance (1, 2). In mammals, RIF1, also known as replication timing regulatory factor 1, functions downstream of the canonical phospho-histone H2A.X-mediator of DNA damage checkpoint 1-tumor protein p53 binding protein 1 (γH2A.X-MDC1-53BP1) signaling axis to influence DNA double-strand break (DSB) repair pathway choice (3). In G_1_ and early S phase, 53BP1-dependent recruitment of RIF1 to DSBs promotes their repair *via* nonhomologous end joining (NHEJ) while suppressing BRCA1 DNA repair associated (BRCA1)-dependent homology-directed repair (HDR) (4, 5, 6, 7). Specifically, RIF1 recruits Shieldin complex (SHLD1-3 and REV7) that suppresses 5′ → 3′ end resection to inhibit HDR (8, 9, 10, 11). RIF1-deficient cells phenocopy elements of 53BP1 deficiency, including NHEJ and class-switch recombination defects, as well as the inappropriate recruitment of BRCA1 to DSBs in G_1_ phase (4, 12, 13, 14). Furthermore, RIF1 deficiency rescues HDR defects and poly(ADP-ribose) polymerase (PARP) inhibitor sensitivity of BRCA1-mutant cells, establishing RIF1 as a key player in 53BP1-dependent DSB repair pathway choice (4, 12).

RIF1 also executes several important roles in DNA replication control. RIF1 is a component of the Bloom’s helicase complex that suppresses deleterious end resection at stalled replication forks to facilitate fork restart and recovery (15). RIF1 also delays replication origin firing through the recruitment of protein phosphatase 1 (PP1) to late origins, leading to dephosphorylation of the minichromosome maintenance complex component 4 (MCM4) of the replicative MCM DNA helicase (16). Therefore, RIF1-deficient cells exhibit delayed DNA replication rates, hypersensitivity to DNA replication inhibitors, and a defect in the ionizing radiation (IR)-induced intra-S phase checkpoint (17, 18).

Possibly independent of its proximal role in suppressing origin firing, RIF1 also regulates genome-wide DNA replication timing through its effects on nuclear architecture, in which it organizes chromatins into topologic domains with similar replication timing in early G_1_ phase prior to the assembly of functional origin recognition complexes (19). RIF1-deficient mammalian cells or yeasts exhibit spatial changes in DNA replication domains that correlate with premature replication origin firing (20, 21, 22) (reviewed in Ref. (23)). RIF1 chromatin occupancy correlates with the replication timing of individual chromatin domains suggesting a role for RIF1 in bundling coregulated origins (24). Interestingly, RIF1 participation in spatial organization of replication domains is genetically separable from its participation in replication timing regulation (25). Finally, RIF1 also plays a role in the resolution of ultrafine DNA bridges in anaphase cells (26, 27).

How RIF1 simultaneously mediates DSB repair pathway choice, replication origin regulation, and nuclear architecture maintenance is unclear. However, it is likely that protein isoforms, modular protein–protein interactions, and pathway-specific posttranslational modifications contribute to RIF1 functional diversity. The amino-terminus of RIF1 (∼1–935 amino acids) comprises an array of 21-folded Huntingtin, elongation factor 3, protein phosphatase 2A alpha subunit, and yeast PI3K TOR1 (HEAT) repeats that exhibits phosphorylation-dependent binding to 53BP1 and binding to SHLD3 (11, 28) (Fig. 1*A*). The partially folded carboxyl-terminal domain (CTD) contains three evolutionarily conserved regions (CR1 – CR3) based on sequence identity among vertebrate RIF1 proteins (15). CR2 additionally bears homology to the CTD of bacterial RNA polymerase α subunits (15). The RIF1 CTD mediates oligomerization, DNA binding, PP1 recruitment (through RVSF motif in CR1), and association with the Bloom’s complex (15, 28, 29, 30, 31, 32) (Fig. 1*A*). However, the full extent of its contributions to various RIF1 functions is unknown.

**Figure 1:**
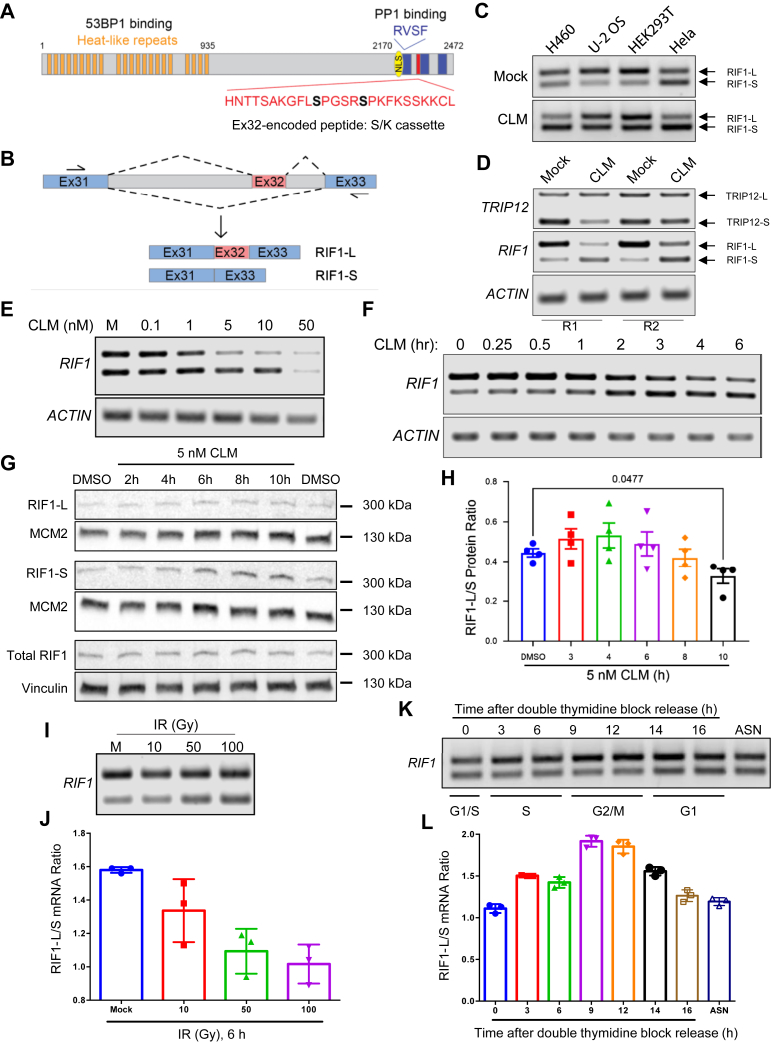
***RIF1* undergoes DNA damage and cell cycle–dependent alternative splicing.***A*, RIF1 protein domains. *Blue rectangles* from *left* to *right* correspond to conserved regions (CR) 1, 2, and 3. Ex32-encoded S/K cassette is highlighted in *red* with its sequence shown. CDK1 phosphorylation sites (S2260 and S2265) are in *bold*. *B*, *RIF1*-Ex32 splicing assay with a forward primer targeting Exon 31 (Ex31) and a reverse primer targeting Exon 33 (Ex33). Exon and intron sizes were drawn in scale relative to each other. *C*, exposure to the radiomimetic drug calicheamicin γ1 (CLM) (10 nM for 6 h) reduced the RIF1-L/RIF1-S mRNA ratio in H460, U-2 OS, HEK293T, and HeLa cells. *D*, differential response of *RIF1* and *TRIP12* alternative splicing to CLM. U-2 OS cells were treated with 10 nM CLM for 6 h, and the total mRNA was analyzed for Ex32 and Ex3 inclusion in RIF1 and TRIP12, respectively. Actin was included as loading control. *E*, CLM dose dependence of *RIF1* splicing regulation in HeLa cells treated with the indicated CLM concentration for 4 h. *F*, time dependence of *RIF1* splicing regulation by 10 nM CLM in U-2 OS cells. *G*, *H*, RIF1-L/RIF1-S protein ratio in HeLa cells treated with 5 nM CLM for the indicated timepoints. The mean RIF1-L/RIF1-S protein ratio ± standard error was calculated by densitometry. Total RIF1, vinculin, and MCM2 were included as loading controls. Each *dot* represents an individual biological replicate, N = 4. Repeated measures one-way ANOVA with Geisser-Greenhouse correction and Dunnett’s multiple comparisons test was performed, and the resulting significant *p*-value was listed. *I*, *J*, dose dependence of ionizing radiation (IR)-induced repression of Ex32 inclusion in H460 cells 6 h after exposure. The mean RIF1-L/RIF1-S mRNA ratio ± standard error was calculated by densitometry. Each *dot* represents an individual biological replicate, N = 3. *K*, *L*, *RIF1* splicing in HeLa cells fluctuates during the cell cycle. HeLa cells were released from a double-thymine block and harvested at the indicated timepoints. The mean RIF1-L/RIF1-S mRNA ratio ± standard error was calculated by densitometry. Each *dot* represents an individual biological replicate, N = 3. CDK1, cyclin-dependent kinase 1; ex, exon; MCM2, minichromosome maintenance complex component 2; RIF1, RAP1 interacting factor 1; RIF1-L, RIF1-Long; RIF1-S, RIF1-Short; S/K cassette, Ser/Lys-rich cassette; TRIP12, thyroid hormone receptor interactor 12.

Here, we show that RIF1 undergoes DNA damage-dependent alternative splicing of Exon 32 (Ex32) to produce RIF1-Long (RIF1-L) and RIF1-Short (RIF1-S) isoforms which differ with respect to a 26 amino acids Ser/Lys-rich (S/K) cassette in the RIF1 CTD. *RIF1* alternative splicing was regulated by a suite of RNA-binding proteins, including serine and arginine rich splicing factor 1 (SRSF1), SRSF3, and SRSF7 whose expression and occupancy on RIF1 pre-mRNA changed in response to DNA damage and in primary cancers. We show that the S/K cassette enhanced RIF1-dependent accumulation of mediator of DNA damage checkpoint 1 (MDC1) at sites of DNA damage; strengthened RIF1 binding to chromatin; and promoted stable phase separation of the RIF1 CTD. By contrast, the DNA damage-induced RIF1-S isoform showed transient phase separation activity and weakly associated with chromatin, suggesting it is a more mobile RIF1 isoform. These studies illuminate mechanisms and functional consequences of *RIF1*-Ex32 splicing in response to DNA damage signaling to execute its roles in maintaining genome integrity.

## Results

### RIF1 undergoes DNA damage and cell cycle–dependent alternative splicing

The human RIF1 is a 2472 amino acids protein of approximately 270 kDa (Fig. 1*A*). RIF1 is expressed as two splice variants, RIF1-S and RIF1-L, that differ by the absence or presence of Ex32. Ex32 encodes a 26 amino acids peptide which we have dubbed the S/K cassette owing to the predominance of Ser and Lys residues (Fig. 1*A*). RT-PCR analysis using primers flanking *RIF1*-Ex32 (Fig. 1*B*) revealed that RIF1-L (the 2472 amino acids isoform) was the major mRNA species in H460, U-2 OS, and HEK293T cells, while HeLa cells had approximately equal amounts of both RIF1 isoforms (Fig. 1*C*). Cellular exposure to the radiomimetic drug calicheamicin γ1 (CLM) increased relative abundance of the RIF1-S (the 2446 amino acids isoform) in all four cell lines suggesting that DNA DSBs repress Ex32 inclusion and promote the formation of RIF1-S (Fig. 1, *C* and *D*). The decreased abundance of RIF1-L mRNA over the same time suggests this isoform may be selectively degraded under conditions of DNA damage. By contrast, the splicing of a cassette exon 3 (Ex3) in the E3 ubiquitin-protein ligase thyroid hormone receptor interactor 12 (TRIP12) was not inhibited following CLM exposure in U-2 OS cells (Fig. 1*D*). Ex32 exclusion and repressed RIF1-L transcript production was CLM dose dependent in HeLa cells (Fig. 1*E*) and reached a maximum at 4 to 6 h after treatment in U-2 OS cells (Fig. 1*F*). As predicted from the changes in *RIF1* splicing, RIF1-L/RIF1-S protein ratio started to decrease at 6 h post-CLM treatment in HeLa cells, associated with an increase in RIF1-S (Fig. 1, *G* and *H*). IR also dose-dependently promoted Ex32 skipping in H460 cells, indicating that Ex32 exclusion is a general response to DNA damage (Fig. 1, *I* and *J*). Finally, *RIF1*-Ex32 splicing also fluctuated during the cell cycle, with the highest RIF1-L/RIF1-S mRNA ratio in G_2_/M-phase and the lowest ratio in G_1_-phase HeLa cells (Fig. 1, *K* and *L*), suggesting that *RIF1* alternative splicing is tightly regulated by cell cycle signaling.

We tested the impacts of canonical DNA repair inhibitors on CLM-dependent Ex32 skipping. While RIF1-L/RIF1-S ratios were comparable between CLM-treated cells with vehicle, PARP, or ATM inhibitors, the combination of CLM and a DNA-PK inhibitor potentiated Ex32 skipping, which may be indicative of enhanced DSB induction (Fig. S1, *A*–*D*). To investigate this, we assessed the extent of H2A.X phosphorylation (γH2A.X) by immunostaining (data not shown) and immunoblotting (Fig. S1, *E* and *F*). However, the expression level of γH2A.X was likely saturated in CLM-treated cells, making it difficult to evaluate the additive effects of the DNA repair inhibitors. We also tested the effects of the transcription inhibitor 5,6-dichloro-1-beta-D-ribofuranosylbenzimidazole (DRB), the topoisomerase I inhibitor camptothecin (CPT), and the DNA replication inhibitor hydroxyurea (HU) on *RIF1*-Ex32 splicing. While DRB and CPT decreased RIF1-L/RIF1-S ratio (Fig. S1, *G* and *H*), HU did not change *RIF1* alternative splicing (Fig. S1, *I* and *J*). These combined findings indicate that *RIF1*-Ex32 splicing is dynamically and specifically regulated by genotoxic stress and during the cell cycle.

### RIF1 isoform usage is altered in cancer

**Figure 2:**
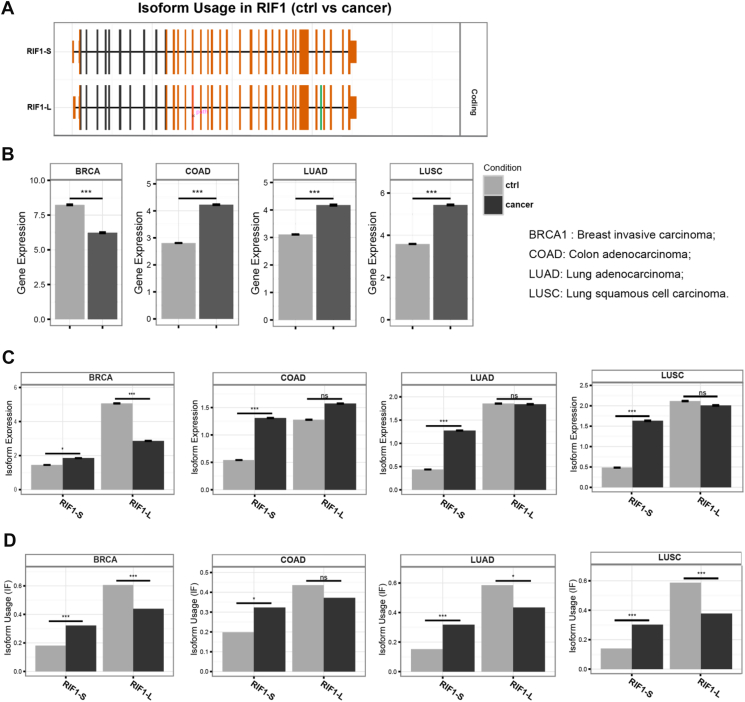
***RIF1*-Ex32 splicing changes across cancer types.***A*, exonic structure of RIF1-S and RIF1-L. *B*, *C*, RIF1 isoform expression data were estimated from The Cancer Genome Atlas (TCGA) data through IsoformSwitchAnalyzeR. *B*, total RIF1 gene expression in breast invasive carcinoma (BRCA) (control, N = 114; cancer, N = 1097), colon adenocarcinoma (COAD) (control, N = 41; cancer, N = 460), lung adenocarcinoma (LUAD) (control, N = 59; cancer, N = 516), and lung squamous cell carcinoma (LUSC) (control, N = 51; cancer, N = 502) (∗∗∗. FDR < 0.001 from EdgeR differential expression analysis and two-tailed Mann–Whitney test). *C*, the proportion of RIF1-S transcript relative to RIF1-L transcript in all four cancer types (ns. not significant; ∗FDR < 0.05; ∗∗∗FDR < 0.001 from EdgeR differential expression analysis and two-tailed Mann-Whitney test). *D*, RIF1 isoform usage (ns. not significant; ∗FDR < 0.05; ∗∗∗FDR < 0.001 from EdgeR differential expression analysis and two-tailed Mann-Whitney test). FDR, false discovery rate; RIF1, RAP1 interacting factor 1; RIF1-L, RIF1-Long; RIF1-S, RIF1-Short.

We queried publicly available RNA-Seq data from The Cancer Genome Atlas to estimate the abundance of RIF1-L and RIF1-S transcripts in normal *versus* tumor tissue across four selected cancer types by IsoformSwitchAnalyzeR (33) (Fig. 2*A*). Total RIF1 mRNA expression was significantly downregulated in breast invasive carcinoma (BRCA) relative to the matched normal breast tissue, while RIF1 expression levels were relatively upregulated in colon adenocarcinoma (COAD), lung adenocarcinoma (LUAD), and lung squamous carcinoma (LUSC) (Fig. 2*B*). When focusing on the isoform levels, RIF1-S mRNA expression was significantly upregulated in BRCA, COAD, LUAD, and LUSC, while RIF1-L expression was only significantly downregulated in BRCA but remained unchanged in COAD, LUAD, and LUSC (Fig. 2*C*). The reduction in RIF1 expression in BRCA could be explained by the reduction in RIF1-L expression whereas the higher RIF1 expression in COAD, LUAD, and LUSC were likely due to the increase in RIF1-S expression. Strikingly, the usage of RIF1-S and RIF1-L isoforms in all four cancer subtypes showed the same trends: RIF1-S isoform usage was significantly increased, while RIF1-L isoform usage was significantly decreased, except in COAD, where RIF1-L isoform usage was slightly reduced, though not statistically significant (Fig. 2*D*). These findings suggest that an RIF1 isoform switch from RIF1-L to RIF1-S may be associated with primary cancers.

### Point mutations in Ex32 disrupt its inclusion into RIF1 transcripts

**Figure 3:**
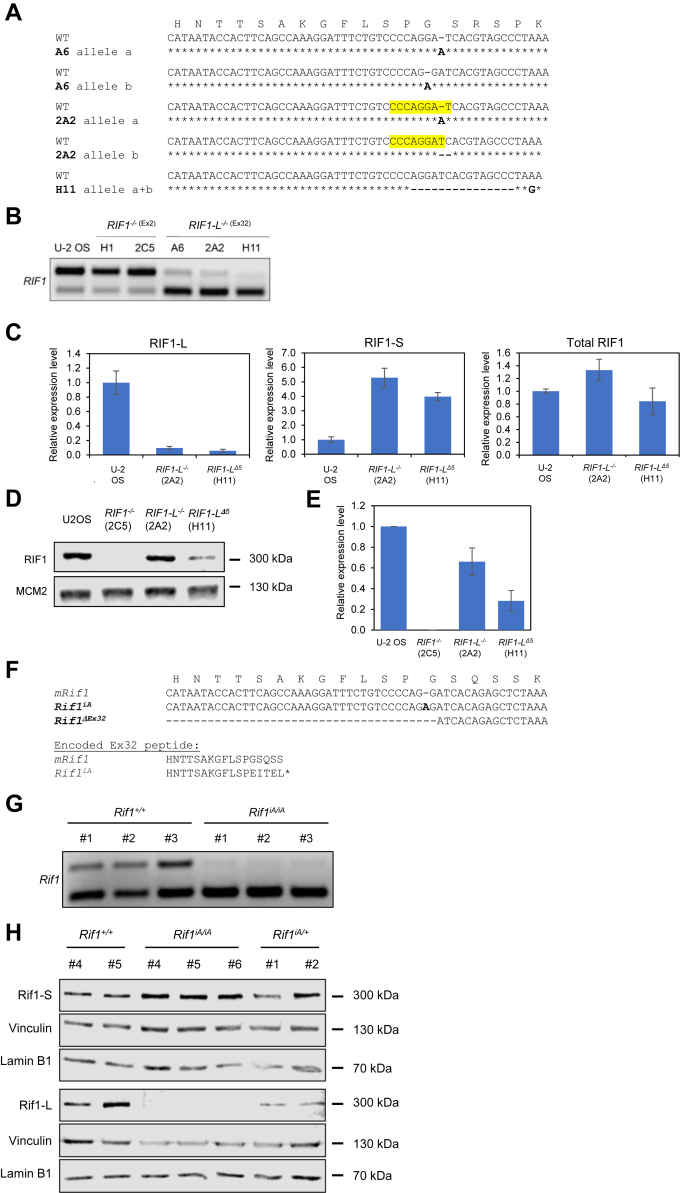
**Point mutations in Ex32 diminish RIF1-L transcript formation.***A*, three *RIF1-L*^*−/−*^ U-2 OS clones (A6, 2A2, and H11) harboring CRISPR/Cas9-induced mutations (in *bold*) aligned to the wildtype (WT) *RIF1*-Ex32 allele. A6 and 2A2 have two distinct alleles “a” and “b”, whereas H11 is homozygous. Disrupted SRSF1-binding site in Clone 2A2 was highlighted in *yellow*. *B*, *RIF1* splicing assay showed that clones A6, 2A2, and H11 produced RIF1-S transcripts predominantly, whereas CRISPR control clones (H1 and 2C5) with guide RNAs targeting *RIF1*-Ex2 and WT U-2 OS cells had more abundant RIF1-L transcripts. *C*, RT-qPCR showed a reduced expression of RIF1-L mRNA and a corresponding increase in RIF1-S mRNA in *RIF1-L*^*−/−*^ U-2 OS cell lines (2A2 and H11). Total RIF1 transcripts stayed relatively constant in WT and mutated U-2 OS cell lines. *D*, *E*, Western blot analysis showed that *RIF1-L*^*−/−*^ (Clone 2A2) and *RIF1-L*^*Δ5*^ (Clone H11) have reduced total RIF1 expression, while Clone 2C5 (*RIF1*^*−/−*^) has undetectable RIF1 expression. MCM2 was included as loading control. *F*, murine *Rif1* alleles generated through CRISPR-mediated gene editing of Ex32. *Rif1*^*iA*^ mice harbor a single A insertion, whereas *Rif1*^*ΔEx32*^ has a 129 nt deletion spanning In31 and Ex32. *G*, *RIF1* splicing assay from three *Rif1*^*+/+*^ and *Rif1*^*iA/iA*^ mice showed reduced expression of RIF1-L and increased expression of RIF1-S mRNA in testis from homozygous *Rif1*^*iA*^ mice. *H*, Western blot analysis of RIF1-L and RIF1-S protein expression in *Rif1*^*+/+*^, *Rif1*^*iA/+*^, and *Rif1*^*iA/iA*^ testis extracts using isoform-specific antibodies showed a similar trend in (*G*). Vinculin and lamin B1 were included as loading controls. CRISPR/Cas9, clustered regularly interspaced short palindromic repeats/Cas9; ex, exon; in, intron; MCM2, minichromosome maintenance complex component 2; RIF1, RAP1 interacting factor; RIF1-L, RIF1-Long; RIF1-S, RIF1-Short; SRSF1, Serine/arginine-rich splicing factor 1.

We used clustered regularly interspaced short palindromic repeats/Cas9 (CRISPR/Cas9) to disrupt Exon 2 (*RIF1*^*−/−*^) or Exon 32 (*RIF1-L*^*−/−*^*)* in U-2 OS cells. We generated two *RIF1*^*−/−*^ lines (H1 and 2C5); two *RIF1-L*^*−/−*^ lines (A6 and 2A2) containing frameshift mutations in Ex32 leading to the creation of a premature stop codon and likely encoded for truncated RIF1-L proteins; and a third *RIF1-L*^*−/−*^ line (H11) harboring a homozygous 15 nt deletion that removes amino acids 2261 to 2265 (Fig. 3*A*). Interestingly, all three *RIF1-L*^*−/−*^ cell lines showed dramatic reductions in RIF1-L transcript and a corresponding increase in RIF1-S transcript while mutations in Exon 2 (Ex2) had no impact on the relative proportion of RIF1-L and RIF1-S transcript (Fig. 3, *B* and *C*). As expected, RIF1-L protein was undetectable in *RIF1-L*^*−/−*^ (2A2) U-2 OS cells, while total RIF1 levels were only slightly reduced relative to controls (Fig. 3, *D* and *E*). On the other hand, *RIF1-L*^*Δ5*^ (H11) harboring an in-frame deletion showed a decrease in both RIF1-L and total RIF1 expression in relative to wildtype U-2 OS cells (Fig. 3, *D* and *E*).

### Point mutations in Ex32 disrupt its inclusion into RIF1 transcripts *in vivo*

We also generated two lines of mice selectively deficient for RIF1-L: a *Rif1*^*iA*^ line harboring a single A insertion between G6604 and G6605 of the RIF1-L coding sequence and *Rif1*^*ΔEx32*^, which harbors a 129 nt deletion spanning the 3′ end of Intron 31 (In31) and the 5′ portion of Ex32 (T6477-G6605) (Fig. 3*F*). Similar to what was observed in U-2 OS cells harboring point mutations in Ex32, expression of RIF1-L mRNA was greatly reduced in testis extracts prepared from *Rif1*^*iA/iA*^ mice, while RIF1-S mRNA was correspondingly upregulated, likely owing to a defect in Ex32 splice-in (Fig. 3*G*). The fact that the iA mutation greatly reduced the RIF1-L/RIF1-S mRNA ratio supports the idea that Ex32 contains an exonic splicing enhancer that is sensitive to small indels. Residual RIF1-L transcripts in *Rif1*^*iA/iA*^ animals contain a premature termination codon in Ex32 and are likely substrates for the nonsense-mediated mRNA decay pathway. As expected, RIF1-L protein was undetectable in homozygous *Rif1*^*iA/iA*^ mice, while RIF1-S protein levels were upregulated relative to *Rif1*^*+/+*^ mice (Fig. 3*H*).

Homozygous *Rif1*^*iA*^ and *Rif1*^*ΔEx32*^ mice were fertile, outwardly normal in appearance, and exhibited normal B and T cell development; normal proportions of mature B and T cells; and comparable rates of mitogen-induced B and T cells proliferation from two independent experiments, each with at least two mice per genotype (Fig. S2, *A*–*F*). In contrast to what was observed in *Rif1*^−/−^ mice (4, 7), B cells from *Rif1*^*ΔEx32*^ mice exhibited normal rates of class switching to IgG1, IgG2a, IgG2b, and IgG3 (Fig. S3, *A*–*D*). Because RIF1-S accounts for all *Rif1* gene dosage in *Rif1*^*ΔEx32*^ mice, these findings indicate that Ex32-encoded S/K cassette is not required for canonical roles of RIF1 in class switch recombination.

### Identification of *RIF1* splicing regulators

**Figure 4:**
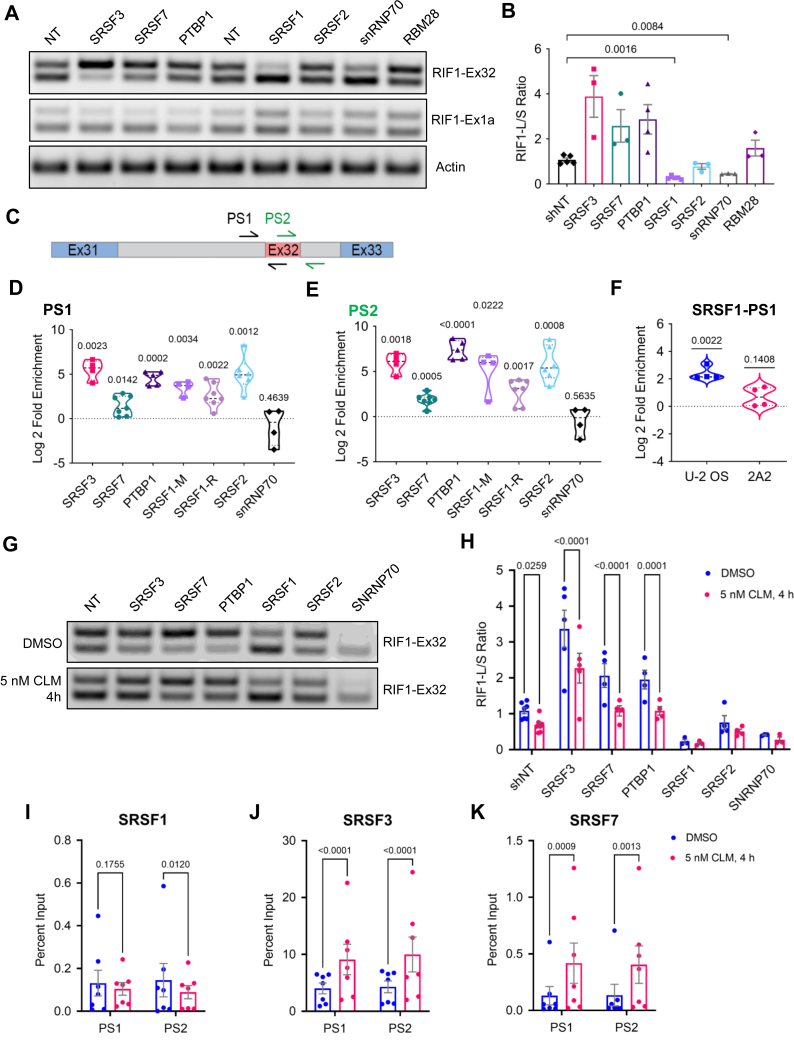

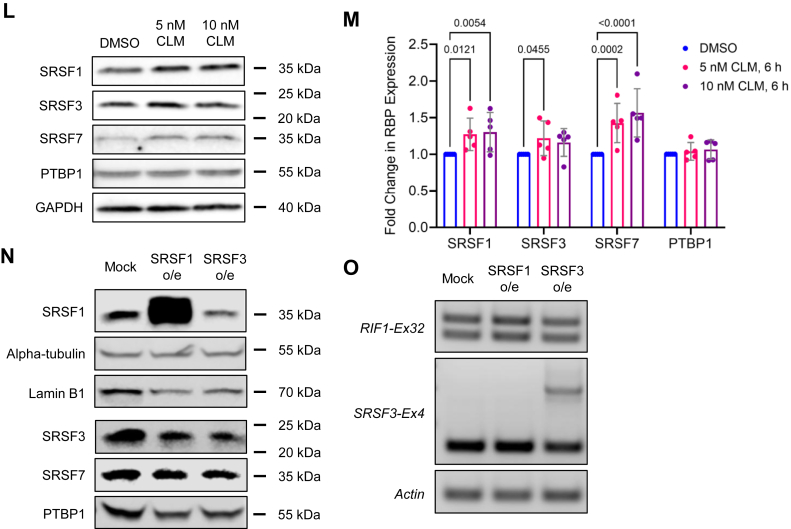
**Identification of RBPs that regulate *RIF1* alternative splicing in response to DNA damage.***A*, lentiviral shRNA vectors targeting putative *RIF1* splicing regulators were transduced into HeLa cells to assess effects on *RIF1*-Ex32 and *RIF1*-Ex1a splicing. Nontargeting (NT) shRNA (shNT) served as a negative control. Actin was included as loading control. *B*, quantification of RIF1-L/RIF1-S mRNA ratios from (*A*) based on densitometry. Each *dot* represents an individual biological replicate, and *bar height* corresponds to mean ± standard error, 3 ≤ N ≤ 5. Brown-Forsythe and Welch ANOVA followed by Dunnett’s T3 multiple comparisons test was performed, and *p*-values from significant comparisons with shNT were shown. *C*, relative locations of the two intron-exon primer pairs used in RNA-IP qPCR experiments. PS1 targets *RIF1*-In31 and Ex32, whereas PS2 targets *RIF1*-Ex32 and In32. *D, E*, relative log_2_ fold enrichment of RIF1 pre-mRNA from native RNA-IP experiments in HeLa cells with antibodies targeting the indicated RBPs from PS1 and PS2 amplification as illustrated in (C). Median and interquartile range were shown as *dotted lines*, and each *dot* represents an individual biological replicate, 4 ≤ N ≤ 7. Two-tailed one sample *t*-test was performed, and the resulting *p*-values were listed. *F*, relative log_2_ fold enrichment of RIF1 pre-mRNA from native RNA-IP experiments done in WT U-2 OS cells and Clone 2A2 (*RIF1-L*^*−/−*^ U-2 OS, Fig. 3*A*) using SRSF1-R antibody. Median and interquartile range were shown as *dotted lines*, and each *dot* represents an individual biological replicate, N = 4. Two-tailed one sample *t*-test was performed, and the resulting *p*-values were listed. *G*, representative gel image of HeLa cells transduced with the indicated lentiviral shRNA vectors and treated with 5 nM CLM for 4 h for *RIF1*-Ex32 splicing assay. *H*, quantification of RIF1-L/RIF1-S mRNA ratios from (G) based on densitometry. Each *dot* represents an individual biological replicate, and *bar height* corresponds to mean ± standard error, 3 ≤ N ≤ 5. Repeated measures two-way ANOVA and Šidák’s multiple comparisons test was performed, and *p*-values were listed for any significant comparisons between DMSO control and CLM-treated groups. *I*–*K*, percent input from native RNA-IP experiments with SRSF1, SRSF3, or SRSF7 antibodies in HeLa cells treated with DMSO or 5 nM CLM for 4 h. Each *dot* represents an individual biological replicate, and *bar height* corresponds to mean ± standard error, N = 7. The *p*-values from repeated measures two-way ANOVA and Šidák’s multiple comparisons test were listed. *L*, representative Western blot image of SRSF1, SRSF3, SRSF7, and PTBP1 expression level from DMSO, 5 nM and 10 nM CLM treatment for 6 h in HeLa cells. Glyceraldehyde-3-phosphate dehydrogenase (GAPDH) was included as loading control. *M*, quantification of the fold change in normalized RBP expression from (*L*) relative to DMSO control. Each *dot* represents an individual biological replicate, and *bar height* corresponds to mean fold change ± standard error, N = 5. Repeated measures two-way ANOVA and Dunnett’s multiple comparisons test was performed, and *p*-values from significant comparisons were listed. *N*, Western blot image showing the overexpression of SRSF1. SRSF3 overexpression likely causes a reduction in the endogenous SRSF3 expression *via* autoregulation. Alpha-tubulin, lamin B1, SRSF7, and PTBP1 were included as loading controls. *O*, overexpression of SRSF1 slightly increased RIF1-L transcripts, while cells transfected with SRSF3 plasmid did not show any changes in *RIF1*-Ex32 splicing but instead induced the inclusion of *SRSF3*-Ex4 through its autoregulation. CLM, calicheamicin γ1; DMSO, dimethyl sulfoxide; ex, exon; in, intron; PS: primer set; PTBP1, polypyrimidine tract-binding protein 1; RBP, RNA-binding protein; RIF1, RAP1 interacting factor 1; RIF1-L, RIF1-Long; RIF1-S, RIF1-Short; RNA-IP, RNA immunoprecipitation; SRSF1, serine and arginine rich splicing factor 1; SRSF3, serine and arginine rich splicing factor 3; SRSF7, serine and arginine rich splicing factor 7; WT: wildtype.

We carried out a small interfering RNA (siRNA) screen of siRNAs targeting 144 RNA-binding proteins (RBPs) for *RIF1* splicing regulators in HeLa cells (Fig. S4, *A*–*C* based on the prediction by RBPmap tool (https://doi.org/10.1093/nar/gku406). The primary siRNA screen and secondary short hairpin RNA (shRNA) validation screen implicated six RBPs whose silencing changed the RIF1-L/RIF1-S splicing ratio at least 2-fold compared to the nontargeting control. Polypyrimidine tract-binding protein 1 (PTBP1), RNA-binding motif protein 28 (RBM28), and SRSFs 3 and 7 were identified as the inhibitors of Ex32 inclusion, whereas small nuclear ribonucleoprotein U1 subunit 70 (snRNP70) and SRSF1 were identified as the enhancers of Ex32 inclusion (Fig. S4, *A*–*C*). The implication of SRSF1 as a *RIF1*-Ex32 splicing enhancer is consistent with findings of Yu *et al.* who identified *RIF1*-Ex32 in a screen for SRSF1-regulated splicing events (34). We investigated SRSF2 as an additional putative enhancer given that its binding site closely overlaps that of SRSF1 (35), and these two splicing factors often work in the same complex with snRNP70 (36). None of the identified *RIF1*-Ex32 splicing regulators significantly modulated *RIF1*-Ex1a splicing in the siRNA screen and shRNA knockdown (Fig. 4*A*).

We next employed native RNA immunoprecipitation (RNA-IP) to test whether candidate *RIF1* splicing regulators directly associated with RIF1 pre-mRNAs. HeLa cell extracts were immunoprecipitated with antibodies specific to SRSF3, SRSF7, PTBP1, SRSF1, SRSF2, and snRNP70 or the corresponding normal mouse and rabbit IgG controls. Following elution and purification, bound pre-mRNA was analyzed by RT-qPCR using two different intron-exon primer pairs, primer set 1 (PS1) and primer set 2 (PS2), as shown in Figure 4*C*. While there was little to no enrichment of *RIF1*-Ex32 containing pre-mRNA from snRNP70-IPs, RNA-IP of all other splicing factors showed significant enrichment over IgG IP controls. In addition, both SRSF1 antibodies used (SRSF1-M and SRSF1-R) showed consistent and significant enrichment, strongly implicating SRSF1 as a direct Ex32 binding factor (Fig. 4, *D* and *E*).

We noted that mutations that disrupted Ex32 inclusion into RIF1 mRNA transcripts in 2A2 and A6 U-2 OS cells were adjacent to a putative SRSF1 binding site (CCCAGGAT) as highlighted in Figure 3*A* (35, 36, 37, 38, 39). Considering this, we compared SRSF1 binding to RIF1 pre-mRNA between wildtype (WT) and *RIF1-L*^*−/−*^ (Clone 2A2) U-2 OS cells. SRSF1 enrichment was not observed in 2A2 U-2 OS cells, suggesting that SRSF1 associates with Ex32 directly through the CCCAGGAT element (Fig. 4*F*).

### DNA damage induces the association of SRSF3 and SRSF7 with RIF1 pre-mRNA

We next sought to determine whether one or more *RIF1*-Ex32 splicing inhibitors were targets of CLM-dependent regulation. We found that the knockdown of any single inhibitor was not sufficient to attenuate Ex32 skipping in CLM-treated HeLa cells, suggesting potential redundant functions between these factors (Fig. 4, *G* and *H*). On the other hand, native RNA-IP demonstrated a significant increase in SRSF3 and SRSF7 association with RIF1 pre-mRNA in CLM-treated HeLa cells (Fig. 4, *J* and *K*). By contrast, SRSF1 association with RIF1 pre-mRNA slightly decreased following CLM treatment, though the effect was marginally significant (Fig. 4*I*). The inverse relationship between SRSF1 and SRSF3/SRSF7 binding to RIF1 transcripts plausibly explains Ex32 skipping in response to CLM. Based on these findings, we evaluated whether CLM affected the localization (Fig. S5*A*) or the expression of SRSF1, SRSF3, SRSF7, and PTBP1 in response to CLM (Fig. 4, *L* and *M*). While the nucleo-cytoplasmic localization of these RBPs did not show any obvious changes after CLM treatment, probably due to the staining of unphosphorylated RBPs (Fig. S5*A*), the total expression of SRSF1 and SRSF7 was significantly increased in CLM-treated HeLa cells (Fig. 4, *L* and *M*). There was also a trend toward higher SRSF3 expression following CLM treatment; however, the difference was only marginally significant (Fig. 4, *L* and *M*). PTBP1 expression was unaffected by CLM.

To further investigate their relationships with *RIF1*-Ex32 splicing, we overexpressed either SRSF1 or SRSF3 in HeLa cells. Overexpression of SRSF1 promoted RIF1-L transcript production as expected (Fig. 4, *N* and *O*). Overexpression of SRSF3 did not affect the RIF1-L/RIF1-S splicing ratio, likely because it promoted the inclusion of a poison cassette exon (Ex4) that negatively regulates SRSF3 levels [Fig. 4, *N* and *O* and Refs (40, 41, 42)].We also queried the expression levels of SRSF1, SRSF2, SRSF3, SRSF7, and PTBP1 in BRCA, COAD, LUAD, and LUSC subsets of The Cancer Genome Atlas data through GEPIA (43). Overexpression of SRSF3 and PTBP1 correlated with the low RIF1-L/RIF1-S ratio associated with these tumors (Figs. 2 and S5*B*). Finally, we also assessed the endogenous expression level of *RIF1* splicing regulators in cell lines which have intrinsically different RIF1-L/RIF1-S mRNA ratios (Figs. 1*C* and S5, C, *D*). While there is a significantly higher SRSF1 expression in osteosarcoma cell lines such as U-2 OS compared to HeLa as previously reported by Li *et al.* (44), we did not observe differences in the expression level of other RBPs.

### RIF1 isoforms behave similarly in DNA replication control

To facilitate study of RIF1-L and RIF1-S isoforms, we reconstituted *RIF1*^*−/−*^ U-2 OS cells with doxycycline (Dox)-inducible, GFP-tagged, RIF1-L, and RIF1-S complementary DNAs (cDNAs) (see Materials and Methods) (Fig. S6*A*). Both *RIF1*^*−/−*^:GFP-RIF1-L and *RIF1*^*−/−*^:GFP-RIF1-S were targeted to IR-induced foci with qualitatively similar magnitude (Fig. S6*B*). GFP-RIF1-L and GFP-RIF1-S comparably suppressed MCM4 hyperphosphorylation, which is reflective of unscheduled replication origin firing (30) (Fig. S6*C*) and rescued the DNA replication patterning defect seen in *RIF1*^*−/−*^ cells. Specifically, RIF1-L and RIF1-S restored the perinuclear and perinucleolar 5-ethynyl-2′-deoxyuridine (EdU) incorporation patterns typical of mid-S phase cells that are almost completely absent in *RIF1*^*−/−*^ cells (Fig. S6, *D*–*F*) (45). Given the basic nature of the S/K cassette and its proximity to the RIF1 DNA-binding domain, we measured DNA binding affinity of purified RIF1-L and RIF1-S C-terminal domains (RIF1^CTD^-L and RIF1^CTD^-S) by fluorescence anisotropy. Binding of RIF1^CTD^-L and RIF1^CTD^-S to an antiparallel G4 substrate was indistinguishable (Fig. S6*G*). Hence, we conclude that these canonical measurements of RIF1 function are not significantly impacted by the S/K cassette.

### Isoform proteomics suggests RIF1 functional differences in genome protection and DSB repair

**Figure 5:**
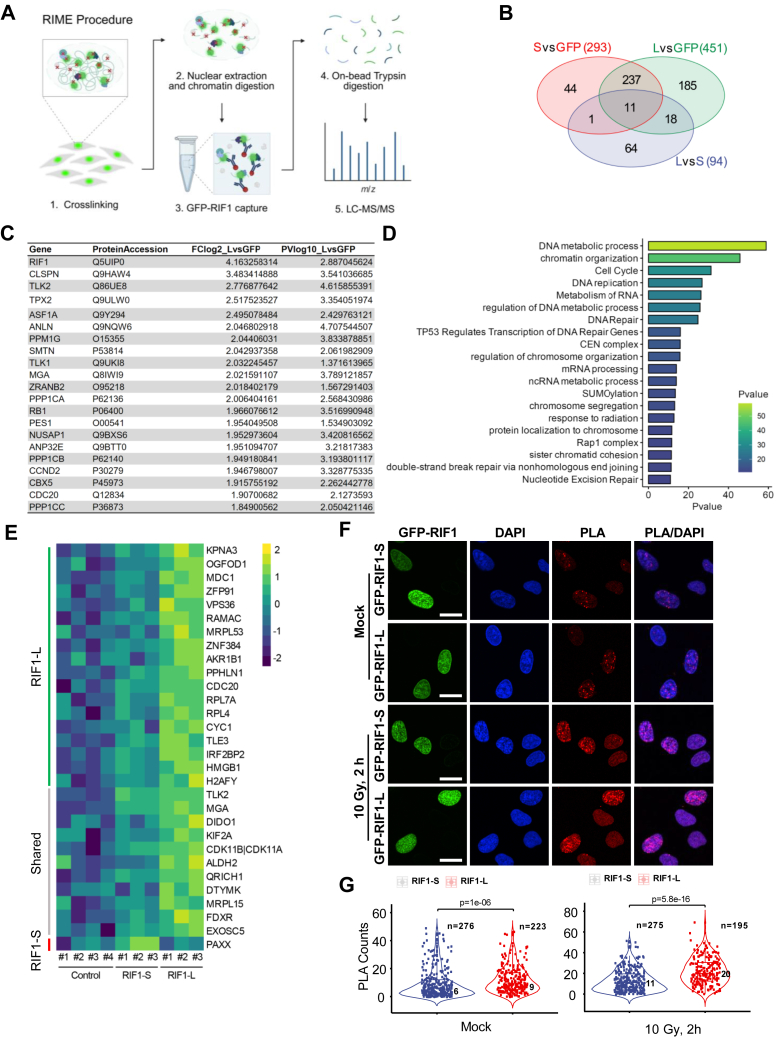

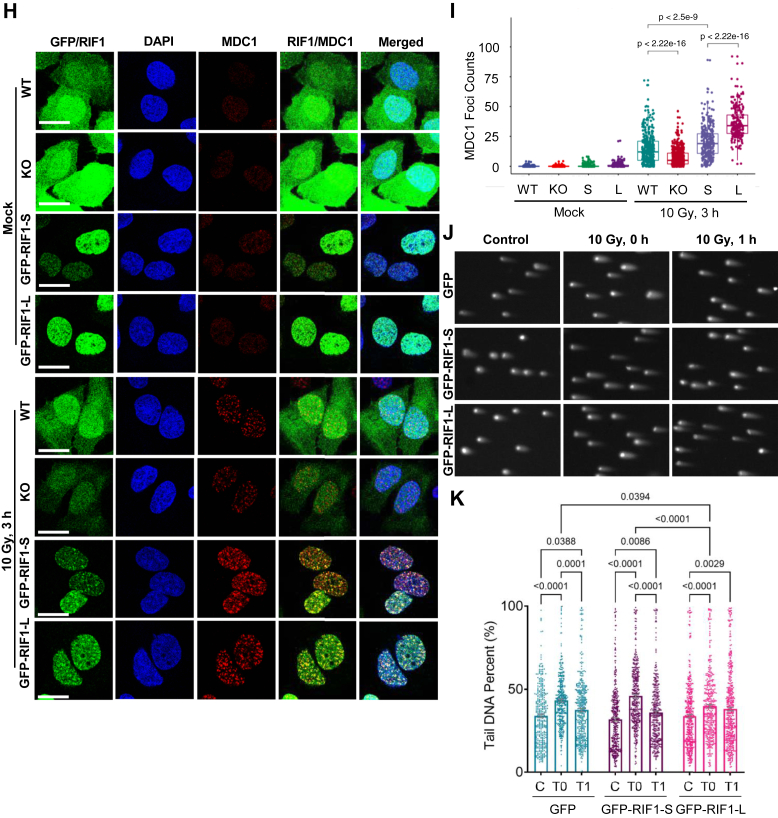
**Chromatin proteomic analysis of RIF1 isoforms showed isoform-specific interactome.***A*, flow chart of the RIF1 RIME procedure. Created in BioRender. Koo, A. (2025) https://BioRender.com/m1wpklr. *B*, Venn diagram showing the number of proteins significantly enriched in GFP-RIF1-S (*red*) and GFP-RIF1-L (*green*) IPs (relative to GFP controls) and proteins differentially enriched between GFP-RIF1-S and GFP-RIF1-L immunoprecipitates (IPs) (*blue*). *C*, top 20 enriched proteins common to GFP-RIF1-S and GFP-RIF1-L datasets. *D*, metascape pathway analysis of the shared GFP-RIF1-S/L interactome. *E*, heat map representation of 30 proteins showing differential enrichment between GFP-RIF1-S and GFP-RIF1-L IPs. *F*, *G*, the interaction between MDC1 and each RIF1 isoforms was evaluated by proximity ligation assay (PLA) using GFP, GFP-RIF1-S, and GFP-RIF1-L *RIF1*^*−/−*^ U-2 OS cells. Cells were processed for PLA using GFP and MDC1 antibodies 2 h after exposure to 10 Gy IR or mock irradiation. The total number of cells analyzed (n), the median number of PLA foci per condition, and the *p*-value from Wilcox test were shown inside each violin plot. Each *dot* represents the PLA count number from an individual cell. Scale bar = 20 μm. *H*, *I*, representative images showed MDC1 foci formation enhanced by RIF1-L. *RIF1*^*+/+*^ (WT), *RIF1*^*−/−*^ (KO), *RIF1*^*−/−*^:GFP-RIF1-S, and *RIF1*^*−/−*^:GFP-RIF1-L U-2 OS cells were mock irradiated or exposed to 10 Gy IR followed by 3 h recovery and then fixed and stained with MDC1 antibodies (Sigma HPA006915, 1:500). WT and KO cells were additionally stained with anti-RIF1 antibodies (Santa Cruz sc515573, 1:100). Foci analysis was performed at a minimum of 50 cells per genotype on CellProfiler. The *p*-values from Wilcox test were shown in the plot. Each *dot* represents the number of MDC1 foci from an evaluated cell. *J*, representative images from neutral comet assays of GFP, GFP-RIF1-S, and GFP-RIF1-L *RIF1*^*−/−*^ U-2 OS cells. Nucleoids from lysed cells exposed to 10 Gy irradiation were collected at time = 0 h or 1 h postirradiation and electrophoresed in neutral TBE buffer. *K*, quantification of the mean comets’ tail DNA percent ± standard error pooled from three biological replicates, each with at least 50 comets scored. Each *dot* represents the tail DNA percent from a nucleoid, total comet number scored ranging from 343 to 439 for each group. Two-way ANOVA with Tukey’s multiple comparisons test was performed, *p*-values from significant comparisons were listed. *C*, control/mock-irradiated cells; T0 = 10 Gy irradiated cells collected at 0 h postirradiation; T1 = 10 Gy irradiated cells collected at 1 h postirradiation. CTD, carboxyl-terminal domain; GFP, green fluorescent protein; IR, ionizing radiation; KO, knockout; MDC1, mediator of DNA damage checkpoint 1; RIF1, RAP1 interacting factor 1; RIF1-L, RIF1-Long; RIF1-S, RIF1-Short; RIME, rapid immunoprecipitation mass spectrometry of endogenous proteins; WT, wildtype.

We carried out quantitative proteomic analysis of GFP-RIF1-L and GFP-RIF1-S stably expressed in *RIF1*^*−/−*^ U-2 OS cells. Because relevant RIF1 interactions are likely to occur in the context of chromatin, we adapted the crosslinking-based RIME (rapid immunoprecipitation mass spectrometry of endogenous proteins) in conjunction with label-free quantitative LC-MS/MS (46) (Fig. 5*A*). We combined data from two technical replicates of three independent RIME crosslinking experiments to identify proteins significantly enriched in α-GFP-RIF1-L and/or α-GFP-RIF1-S immunoprecipitates (IPs) *versus* α-GFP IPs (Table S2). Using an false discovery rate (FDR) of < 0.05, 451 proteins were significantly enriched in GFP-RIF1-L. A total of 293 proteins were identified in GFP-RIF1-S IPs, of which, 248 were also identified for GFP-RIF1-L (Fig. 5*B*). The reduced number of interactants for GFP-RIF1-S may reflect reduced chromatin association (see below) or its slightly lower expression in *RIF1*^*−/−*^ U-2 OS cells (Fig. S6*A*).

The dataset of shared RIF1-interacting proteins contained known RIF1 interactors, including PP1, 53BP1, the serine/threonine-protein kinase tousled-like 2 (TLK2) implicated in nucleosome assembly, DNA replication, and DNA repair, and the anti-silencing function 1A histone chaperone ASF1A (47, 48, 49, 50, 51) (Fig. 5*C*) as well as factors not previously reported to associate with RIF1. Outside of RIF1 itself, the most highly enriched protein in RIF1-L and RIF1-S IPs was Claspin (CLSPN), an adaptor protein that facilitates ataxia telangiectasia and Rad3-related protein (ATR)-dependent activation of the effector checkpoint kinase 1 (CHK1) in response to DNA replication inhibition (52, 53). Other novel RIF1-associated proteins identified in both RIF1-S and RIF1-L IPs include DNA topoisomerase II alpha (TOP2A) and several proteins involved in mitosis, including microtubule nucleation factor TPX2, kinesin family member 4A (KIF4A), kinesin family member 23 (KIF23), and centromere protein F (CENPF) (54, 55, 56, 57, 58); the histone chaperone acidic nuclear phosphoprotein 32 family member E (ANP32E) (59); the telomerase-associated pescadillo ribosomal biogenesis factor 1 (PES1) (60); and the mitotic checkpoint regulator cell division cycle 20 (CDC20) (61, 62) (Fig. 5*C*). Metascape analysis identified DNA metabolic process, chromatin organization, cell cycle, DNA replication, and DNA repair as overrepresented gene functional groups in RIF1-L/RIF1-S chromatin proteomes (Fig. 5*D*).

A comparison of the GFP-RIF1-L and GFP-RIF1-S RIME datasets yielded a total of 94 differentially enriched proteins (Fig. 5*B*); however, 64 of these were excluded from further analysis because they were not significantly enriched in either GFP-RIF1-L or GFP-RIF1-S IPs relative to GFP controls. Of the remaining 30 proteins, 11 were detected in both GFP-RIF1-L and GFP-RIF1-S but were more enriched in GFP-RIF1-L; 18 proteins were selectively enriched in GFP-RIF1-L IPs; and one protein, the NHEJ regulator PAXX (paralog of XRCC4 and XLF) (63), was selectively enriched in GFP-RIF1-S IPs (Fig. 5*E*).

The two proteins showing the greatest fold-change difference between GFP-RIF1-L and GFP-RIF1-S IPs were the nuclear import receptor karyopherin α 3 (KPNA3) and MDC1 (Fig. 5*E*, Table S2). The apparent ∼4-fold enrichment of MDC1 in RIF1-L IPs is consistent with a study by Gupta *et al.* that identified RIF1 peptides in MDC1 proximity labeling studies (9) and was particularly interesting given that MDC1 recruits RNF8 and consequently 53BP1 and RIF1 to the sites of DNA damage (5, 55, 56, 57, 64, 65, 66). In support of the RIME-MS analysis, a proximity ligation assay (10.13039/100009437PLA) revealed that endogenous MDC1 was associated with both RIF1-L and RIF1-S and that the number of PLA foci was significantly greater in GFP-RIF1-L *versus* GFP-RIF1-S U-2 OS cells (Fig. 5, *F* and *G*). This interaction was further strengthened upon irradiation (Fig. 5, *F* and *G*). Given these findings, we evaluated IR-induced MDC1 focus formation in *RIF1*^*+/+*^, *RIF1*^*−/−*^, *RIF1*^*−/−*^:GFP-RIF1-L, and *RIF1*^*−/−*^:GFP-RIF1-S U-2 OS cells. The number of MDC1 foci was significantly reduced *in RIF1*^*−/−*^ cells relative to *RIF1*^*+/+*^ cells 3 h after exposure to IR, suggesting RIF1 enhances stable MDC1 recruitment (Fig. 5, *H* and *I*). Furthermore, MDC1 foci were more abundant in GFP-RIF1-L U-2 OS cells *versus* GFP-RIF1-S U-2 OS cells, suggesting that RIF1-L amplifies MDC1 accumulation at DSBs.

The selective enrichment of PAXX in GFP-RIF1-S RIME IPs suggests a direct link between RIF1-S and NHEJ. Because PAXX participates in NHEJ through interaction with Ku70 and promoting DSB end synapsis (63, 67, 68, 69, 70, 71, 72), we tested RIF1-L and RIF1-S cells for global DSB repair activity using a neutral comet assay, which primarily measures the liberation of double-stranded DNA fragments from permeabilized nuclei. Initial levels of DSBs were significantly lower in GFP-RIF1-L cells *versus* GFP-RIF1-S and GFP *RIF1*^*−/−*^ U-2 OS cells immediately following exposure to 10 Gy IR at T0, suggesting a potential role in genome protection for RIF1-L (Fig. 5, *J* and *K*). The levels of residual DSBs an hour post-IR (T1) were comparable between GFP-RIF1-L and GFP-RIF1-S cells but slightly elevated in GFP controls (Fig. 5, *J* and *K*). Thus, while GFP-RIF1-S cells were more prone to IR-induced DSB, the steeper slope between T0 and T1 suggests an enhanced rate of DSB repair in GFP-RIF1-S *versus* GFP-RIF1-L cells. We also employed NHEJ and HDR reporter assays using wildtype HEK293T cells (which express mostly RIF1-L) and two *RIF1-L*^*−/−*^ clones (#1 and #2) (Fig. S6, *H*–*J*). Both *RIF1-L*^*−/−*^ lines showed reduced repair of the NHEJ and HDR substrates, potentially due to a defect in sustained MDC1 recruitment to DSBs. Reduced cycling rates of the *RIF1-L*^*−/−*^ HEK293T clones may have also contributed to the apparent reduction in HDR efficiency (not shown).

### CTD phosphorylation diminishes RIF1 chromatin and MDC1 association

**Figure 6:**
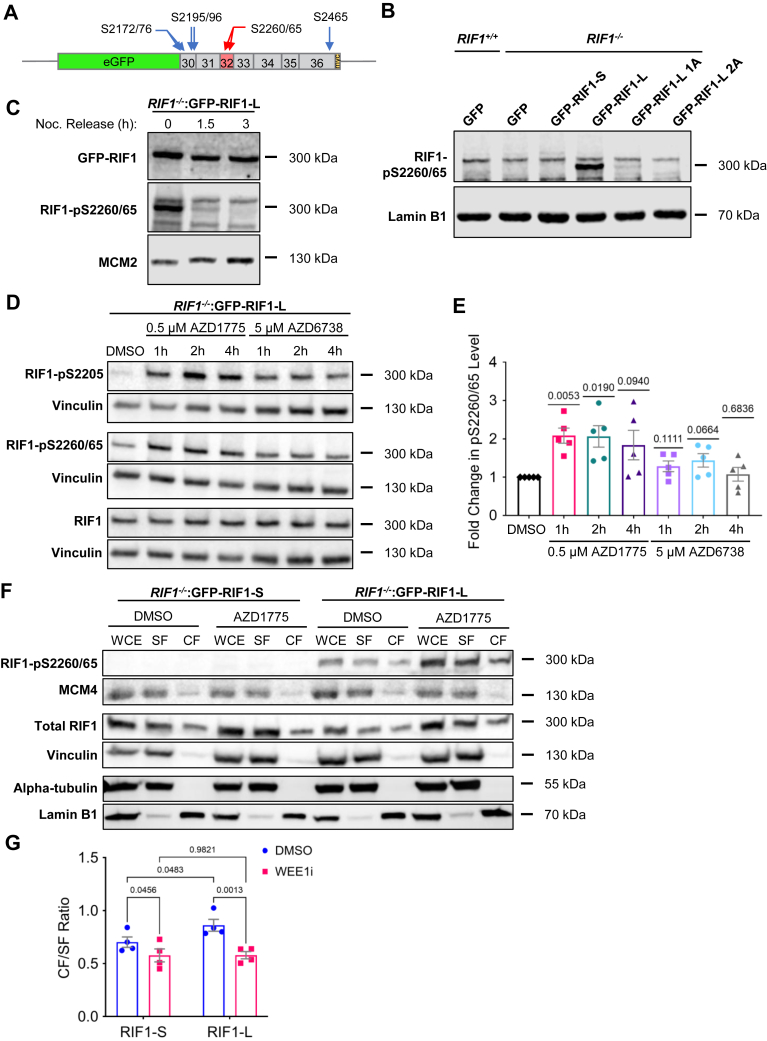

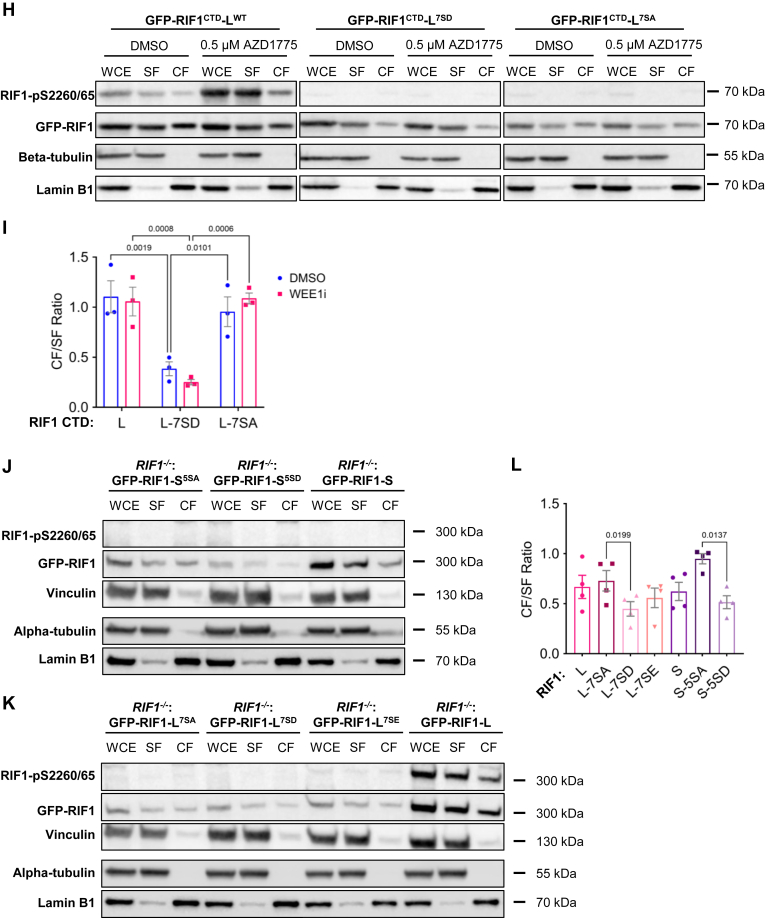

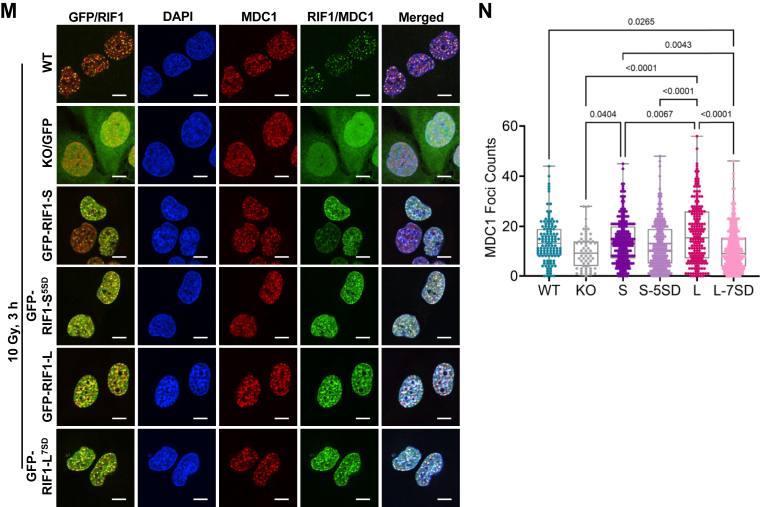
**RIF1 phosphorylation on S2260 and S2265 during mitosis and in response to WEE1 inhibitordecreases its chromatin association.***A*, schematic of GFP-RIF1^CTD^-L showing S2260/65 (*red arrows*) and five additional UniProt-annotated phospho-Ser residues (*blue arrows*). These selected Ser residues were subsequently mutated to Asp or Ala. *B*, specificity of α-RIF1-pS2260/65 antisera was tested with lysates from *RIF1*^*−/−*^ U-2 OS cells expressing GFP or the indicated RIF1 alleles. RIF1-L^1A^ and RIF1-L^2A^ alleles harbor Ala mutations at S2260 and S2260/S2265, respectively. Lamin B1 was included as loading control. *C*, *RIF1*^*−/−*^:GFP-RIF1-L U-2 OS cells were synchronized in prometaphase with nocodazole (Noc) and released into Noc-free media for the indicated lengths of time. Note rapid reduction in RIF1-pS2260/65 levels following Noc release. GFP-RIF1 and MCM2 were included as loading controls. *D*, GFP-RIF1-L U-2 OS cells were treated with WEE1 inhibitor (0.5 μM AZD1775) or ATR inhibitor (5 μM AZD6738) for the indicated timepoints prior to immunoblotting analysis with the indicated antibodies. Total RIF1 and vinculin were included as loading controls. *E*, quantification of RIF1-pS2260/65 level from (*D*) based on densitometry. *Bar height* represents mean fold change in RIF1-pS2260/65 relative to the baseline phosphorylation level in DMSO control ± standard error. Each *dot* represents an individual biological replicate, N = 5. The *p*-values from two-tailed one sample *t*-test were listed. *F*, representative Western blot image showing the chromatin fractionation patterns for GFP-RIF1-L and GFP-RIF1-S cells treated with DMSO or 0.5 μM AZD1775 for 1 h. Whole-cell extract (WCE), chromatin fraction (CF), and soluble fraction (SF) were resolved by SDS-PAGE and blotted with the indicated antibodies. Vinculin, alpha-tubulin, and lamin B1 were included as loading and fractionation controls. *G*, quantification of the mean chromatin/soluble fraction (CF/SF) ratio of total RIF1-L/RIF1-S ± standard error based on densitometry. Each *dot* represents an individual biological replicate, N = 4. Repeated measures two-way ANOVA with uncorrected Fisher’s LSD test was performed, and the resulting *p*-values were listed. *H*, chromatin fractionation patterns of GFP-RIF1^CTD^-L and its serine mutants. The indicated GFP-RIF1^CTD^-L constructs were stably transfected into U-2 OS cells. The cells were treated with DMSO or 0.5 μM AZD1775 for an hour for chromatin fractionation assay. Note that phospho-GFP-RIF1^CTD^-L^WT^ and phosphomimetic GFP-RIF1^CTD^-L^7SD^ were highly enriched in the SF compared to the corresponding CF, while GFP-RIF1^CTD^-L^7SA^ with abolished phosphorylation sites was not. Beta-tubulin and lamin B1 were included as loading and fractionation controls. *I*, quantification of the mean CF/SF ratio of GFP-RIF1 in (H) ± standard error based on densitometry. Each *dot* represents an individual biological replicate, N = 3. Two-way ANOVA with Tukey’s multiple comparisons test was performed, and the *p*-values from significant comparisons were listed. *J*, *K*, chromatin fractionation patterns of full-length GFP-RIF1 isoforms and its serine mutants. *RIF1*^*−/−*^ U-2 OS cells expressing the indicated GFP-RIF1-S (J) and GFP-RIF1-L (*K*) constructs were subjected to chromatin fractionation. Vinculin, alpha-tubulin, and lamin B1 were included as loading and fractionation controls. *L*, quantification of the mean CF/SF ratio of GFP-RIF1 in (*J*) and (K) ± standard error based on densitometry. Each *dot* represents an individual biological replicate, N = 4. Repeated measures one-way ANOVA with Geisser–Greenhouse correction and Tukey’s multiple comparisons test was performed, and the *p*-values from significant comparisons were shown. *M*, representative MDC1 foci images of *RIF1*^*+/+*^ (WT), *RIF1*^*−/−*^:GFP (KO/GFP), *RIF1*^*−/−*^:GFP-RIF1-S, *RIF1*^*−/−*^:GFP-RIF1-S^5SD^, *RIF1*^*−/−*^:GFP-RIF1-L, and *RIF1*^*−/−*^:GFP-RIF1-L^7SD^ U-2 OS cells after 10 Gy irradiation followed by 3 h recovery before being stained with MDC1 antibodies (Sigma HPA006915, 1:500). WT cells were additionally stained with anti-RIF1 antibodies (Santa Cruz sc515573, 1:100). Scale bar = 10 μm. *N*, box plots showing the quantification of MDC1 foci counts per genotype by ImageJ macro Foci_Analyzer_1_5. Each *dot* represents the number of MDC1 foci from an evaluated cell, and total cells scored ranging from 59 to 277 for each genotype. One-way ANOVA with Tukey's multiple comparisons test was performed, and the *p*-values from any significant comparisons were listed. ATR, ataxia telangiectasia and Rad3-related protein; DMSO, dimethyl sulfoxide; GFP, green fluorescent protein; KO, knockout; MDC1, mediator of DNA damage checkpoint 1; RIF1, RAP1 interacting factor 1; RIF1-L, RIF1-Long; RIF1-S, RIF1-Short; WT: wildtype.

Orbitrap MS identified several phosphorylation sites in GFP-RIF1^CTD^-L, including S2205, which is located in the PP1 binding site; S2260 and S2265, which are located in Ex32-encoded S/K cassette (Fig. 6*A*); and S2348, which lies within CR2. We generated phospho-specific antibodies against a peptide dually phosphorylated on S2260 and S2265 (α-RIF1-pS2260/65) and validated the site specificity of the antibody in Western blotting experiments using GFP-RIF1-L^1A^ and GFP-RIF1-L^2A^ mutants with Ala mutation at site(s) S2260 and S2260/65. GFP-RIF1-L^1A^ reduced α-RIF1-pS2260/65 recognition dramatically, while GFP-RIF1-L^2A^ completely abolished recognition by this antibody (Fig. 6*B*). Using nocodazole-synchronized U-2 OS cells, we found that RIF1-pS2260/65 phosphorylation was maximal in mitosis and rapidly extinguished following mitotic exit (Fig. 6*C*).

S2260 and S2265 residues occur in a Ser-Pro dipeptide motif that is a consensus for the mitotic cyclin-dependent kinase 1 (CDK1) and related kinases. A recent study suggested that, under conditions of ATR inhibition, CDK1-dependent phosphorylation of RIF1 on S2205 diminished PP1 binding, leading to increased phosphorylation stoichiometry of cyclin-dependent kinase 2 (CDK2) and cell division cycle 7 (CDC7) substrates and elevated rates of origin firing (73, 74). To determine whether S2260 and S2265 phosphorylation exhibit a similar phosphorylation profile, we measured RIF1-pS2260/65 levels in GFP-RIF1-L U-2 OS cells cultured in the presence of a WEE1 G_2_ checkpoint kinase inhibitor (WEE1i, AZD1775) or an ATR inhibitor (ATRi, AZD6738). AZD1775 treatment significantly increased RIF1-pS2260/65 level in the first 2 h after treatment, while ATR inhibitors had little effect (Fig. 6, *D* and *E*). Thus, RIF1-S2205 and RIF1-S2260/65 phosphorylation sites are cophosphorylated under conditions of aberrant CDK1 activation. We also examined the effect of HU-induced replication stress on RIF1-S2260/65 phosphorylation using the same cell line but found a slight reduction in RIF1-pS2260/65 (Fig. S7, *A* and *B*).

The basic nature of the S/K cassette suggested it may play a role in nuclear localization, DNA binding, and/or chromatin association. While RIF1-L and RIF1-S exhibited comparable nuclear localization and binding to G-quadraplex DNA substrates (Fig. S6, *B* and *G*), chromatin fractionation of full-length GFP-RIF1-L and GFP-RIF1-S suggested that RIF1-L has significantly higher chromatin-binding affinity relative to RIF1-S (Fig. 6, *F* and *G*, *blue bars*). WEE1 inhibition using AZD1775 increased the proportion of S2260/65-phosphorylated RIF1-L and decreased the chromatin association of both RIF1-L and RIF1-S (Fig. 6, *F* and *G*, *red bars*).

To further explore the relationship between RIF1 phosphorylation and its chromatin association, we compared the chromatin association profiles of GFP-RIF1^CTD^-L^WT^ to that of GFP-RIF1^CTD^-L^7SA^ and GFP-RIF1^CTD^-L^7SD^ mutants harboring seven Ser-Ala (SA) or Ser-Asp (SD) mutations at UniProt-annotated CDK1 phosphorylation sites, including S2260 and S2265, within the S/K cassette (Fig. 6*A*). Similar to what was observed using full-length RIF1-L, the phosphorylation of RIF1^CTD^-L^WT^ on S2260/65 was significantly increased in response to WEE1 inhibition with AZD1775 (Fig. 6*H*, *left panel*). In addition, the RIF1-pS2260/65 signal was strongly enriched in the soluble fraction (SF) relative to the chromatin fraction, suggesting that phosphorylation reduces RIF1^CTD^ chromatin-binding affinity. Consistent with this, the phosphomimetic RIF1^CTD^-L^7SD^ mutant exhibited a significant reduction in chromatin association even without AZD1775 treatment (Fig. 6*H*, *center panel* and Fig. 6*I*). While the RIF1^CTD^-L^7SA^ mutant exhibited lower expression, its chromatin-binding profile was similar to RIF1^CTD^-L^WT^ (Fig. 6*H*, *right panel* and Fig. 6*I*). As expected, Western blotting with RIF1-pS2260/65 antibodies did not yield a signal in U-2 OS cells expressing either RIF1^CTD^-L^7SD^ or RIF1^CTD^-L^7SA^. Based on these findings, we reconstituted *RIF1*^*−/−*^ U-2 OS cells with full-length GFP-RIF1-S^5SA^, GFP-RIF1-S^5SD^, GFP-RIF1-L^7SA^, GFP-RIF1-L^7SD^, and GFP-RIF1-L^7SE^ constructs. Consistent with what was observed with GFP-RIF1^CTD^ fragments, abolishing phosphorylation sites by SA mutations increased chromatin retention significantly compared to the phosphomimetic SD and Ser-Glu (SE) mutants (Fig. 6, *J*–*L*). Altogether, findings with full-length RIF1 and RIF1^CTD^ fragments suggest that multiple phosphorylation sites within the RIF1 CTD, including S2260/2265 in the S/K cassette, diminish RIF1 chromatin binding in response to WEE1 inhibition.

Since the RIF1-L-MDC1 association occurred in the context of chromatin (Fig. 5), we asked whether RIF1 CTD phosphorylation site mutants showed any defect in MDC1 focus formation. Consistent with findings in Figure 5, *H* and *I*, GFP-RIF1-L rescued the MDC1 focus formation defect of *RIF1*^*−/−*^ cells to a greater extent than GFP-RIF1-S U-2 OS cells (Fig. 6, *M* and *N*). The GFP-RIF1-L^7SD^ mutant showed significantly fewer MDC1 foci *versus* GFP-RIF1^WT^-L. From this, we surmise that phosphorylation of the S/K cassette and flanking CTD diminishes RIF1 association with MDC1 on chromatin.

We also noted RIF1-S2265 falls within a BRCT (BRCA1 C-terminal) binding motif (SPxF). While AlphaFold 3 (75) predicted interactions between RIF1-pS2260/65 residues with MDC1-R1930 and E1974 which lie within the MDC1-BRCT domain (Fig. S7*C*), this domain has been shown to specifically interact with phospho-H2A.X C-terminal domain (76). *RIF1*^*−/−*^ cells did not exhibit a defect in MDC1 foci formation on mitotic chromosomes in nocodazole-arrested cells (not shown), while both RIF1-L and RIF1-S were evicted from mitotic chromosomes (Fig. S8). Based on the finding that RIF1-pS2260/65 was induced during mitosis (Fig. 6*C*) and RIF1 is evicted from mitotic chromosomes (Fig. S8), we concluded that RIF1-L-MDC1 interaction functions outside of the mitotic phase.

### The S/K cassette regulates RIF1 phase separation

**Figure 7:**
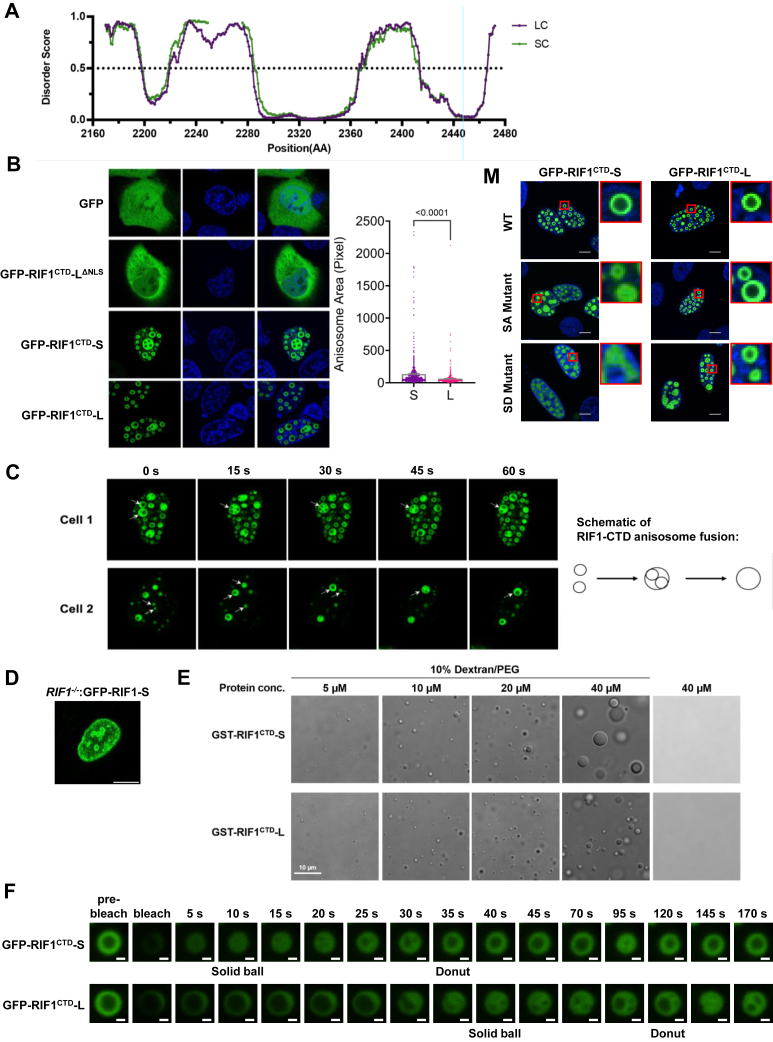

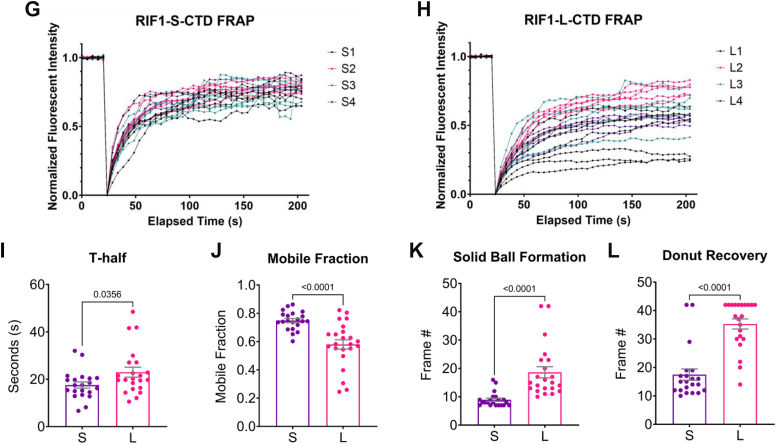
**The S/K cassette stabilizes phase separation of the RIF1 CTD.***A*, predictive disorder score of GFP-RIF1^CTD^-S and GFP-RIF1^CTD^-L showed that the presence of S/K cassette (2250–2275 amino acids) decreases the disorder of RIF1 CTD. *B*, (*left panel*) representative examples of RIF1 CTD anisosomes. U-2 OS cells were transiently transfected with GFP, GFP-RIF1^CTD^-L^ΔNLS^, GFP-RIF1^CTD^-S, or GFP-RIF1^CTD^-L and subjected to live cell imaging after Hoechst 33342 staining (*blue*). *B*, (*right panel*) quantification of the mean anisosome area ± standard error by customized ImageJ script. Each *dot* represents an anisosome scored, n = 586 for GFP-RIF1^CTD^-S; n = 396 for GFP-RIF1^CTD^-L for the total number of anisosomes. The *p*-value from unpaired two-tailed *t-*test assuming equal standard deviation was shown. *C*, timelapse images and the schematic showing the fusion of GFP-RIF1^CTD^ anisosomes in 60 s. Single anisosomes and the subsequently fused multichambers anisosomes were marked with *white arrows*. *D*, full-length GFP-RIF1-S occasionally forms anisosome-like structure in Dox-inducible *RIF1*^*−/−*^ U-2 OS cells. Scale bar = 10 μm. *E*, *in vitro* phase separation assays showed concentration (10–40 μM)-dependent LLPS droplet formation of purified GST-RIF1^CTD^-S and GST-RIF1^CTD^-L proteins. Scale bar = 10 μm. *F*, fluorescence recovery after photobleaching (FRAP) montage for representative GFP-RIF1^CTD^-S and GFP-RIF1^CTD^-L anisosomes in U-2 OS cells. Morphological distinct anisosome structures—“Solid ball” and “Donut”—were depicted. Scale bar = 1 μm. *G*, *H*, FRAP recovery curves of the bleached anisosomes for GFP-RIF1^CTD^-S and GFP-RIF1^CTD^-L over a time course of 3 minutes. Four biological replicates were carried out, each with at least three technical replicates. Each recovery curve was color-coded according to the biological replicate number (S1-4 or L1-4) in each *plot*. *I*, *J*, each recovery curve from (*G*) and (*H*) was fitted by single exponential equation to estimate the t-half value of recovery and mobile fraction of the anisosome. *Bar height* represents mean ± standard error of the pooled data. Each *dot* represents score from an anisosome, n = 21 for GFP-RIF1^CTD^-S and n = 22 for GFP-RIF1^CTD^-L. The *p*-values from unpaired two-tailed *t*-tests with Welch’s correction which do not assume equal standard deviation in populations were listed. *K*, *L*, manual tabulation of the recovery frame where the two distinct stages—“solid ball” and “donut”, as indicated in (*F*)—reappeared after photobleaching was done. *Bar height* represents mean ± standard error of the pooled data. Each *dot* represents score from an anisosome, n = 21 for GFP-RIF1^CTD^-S and n = 22 for GFP-RIF1^CTD^-L. The *p*-values from unpaired two-tailed *t*-tests with Welch’s correction were shown. *M*, representative images showed that serine to alanine (SA) and serine to aspartic acid (SD) mutations impede anisosome formation to a greater extent in GFP-RIF1^CTD^-S expressing cells. *Red boxes* showed enlargement of the anisosomes of interest. Scale bar = 10 μm. CTD, carboxyl-terminal domain; GFP, green fluorescent protein; GST, glutathione-S-transferase; LLPS, liquid–liquid phase separation; RIF1, RAP1 interacting factor 1; RIF1-L, RIF1-Long; RIF1-S, RIF1-Short; S/K cassette, Ser/Lys-rich cassette.

Using the DISOPRED 3.1 disorder prediction tool (77), we found that the presence of the S/K cassette reduced disorder of the region roughly spanning amino acids ∼2240 to 2280 in the RIF1 CTD (Fig. 7*A*). In transient transfection assays, GFP-RIF1^CTD^-L and GFP-RIF1^CTD^-S formed spherical shells in the nuclei of U-2 OS cells that ranged from single shells to complex arrangements containing multiple chambers (Fig. 7, *B* and *C*). In contrast, GFP-RIF1^CTD^-L^ΔNLS^ with a deletion of the nuclear localization signal showed diffused GFP signal in the cytoplasm (Fig. 7*B*). Three-dimensional reconstruction revealed RIF1 nuclear shells to be oblong spheroids, each with a hollow central core (Video S1). Because they closely resembled the birefringent “anisosomes” formed by the nuclear RNA-binding protein TDP-43 (78), we have adopted the anisosome nomenclature to describe RIF1^CTD^ nuclear assemblies.

Time lapse imaging revealed RIF1^CTD^ nuclear anisosomes to be dynamic structures that frequently fused to form larger single- or multi-chamber structures (Fig. 7*C*, Video S2). In addition, GFP-RIF1^CTD^ anisosomes underwent spontaneous cycles of closure and reopening (Video S2). While both GFP-RIF1^CTD^-S and GFP-RIF1^CTD^-L formed anisosomes, they exhibited different properties. GFP-RIF1^CTD^-S anisosomes exhibited an increased rate of fusion events *versus* GFP-RIF1^CTD^-L anisosomes to form larger structures (Fig. 7*B*), while GFP-RIF1^CTD^-L anisosomes occasionally formed nested structures—rarely observed for GFP-RIF1^CTD^-S—in which smaller anisosomes were enclosed within a larger assembly (Video S3). These nested anisosomes may be fusion intermediates and were therefore observed more frequently in the less dynamic GFP-RIF1^CTD^-L which fused slower. RIF1^CTD^ nuclear anisosomes were also observed in transiently transfected HeLa and HEK293T cells as well as U-2 OS cells expressing Dox-inducible GFP-RIF1^CTD^-S and GFP-RIF1^CTD^-L (not shown). Full-length GFP-tagged RIF1 protein, on rare occasion, can also form anisosome-like droplets as shown in Figure 7*D*.

The assembly of GFP-RIF1^CTD^ into anisosomes suggested the CTD undergoes liquid–liquid phase separation (LLPS). To test this, we incubated purified, glutathione-S-transferase (GST)-tagged RIF1^CTD^-S and RIF1^CTD^-L with 10% dextran/polyethylene glycol. Both GST-RIF1^CTD^-S and GST-RIF1^CTD^-L underwent concentration-dependent LLPS; however, RIF1^CTD^-S formed significantly larger droplets compared to RIF1^CTD^-L with increasing RIF1^CTD^ concentration (Fig. 7*E*), possibly reflecting increased rates of droplet fusion seen in transient transfection assays (Fig. 7*C*).

To better evaluate the dynamics of RIF1^CTD^ anisosomes, we performed fluorescence recovery after photobleaching (FRAP) experiments in which GFP-RIF1^CTD^-S and GFP-RIF1^CTD^-L anisosomes were allowed to recover after photobleaching. Both GFP-RIF1^CTD^-S and GFP-RIF1^CTD^-L anisosomes rapidly disintegrated following photobleaching, losing their donut-like character with laser exposure before reassembling over the course of 3 min (Fig. 7*F*, time course/montage, Video S4-S5). The recovery curve of each bleached anisosome was then plotted from the average fluorescent intensity of the bleached region over time (Fig. 7, *G* and *H*). GFP-RIF1^CTD^-S anisosomes, when compared to GFP-RIF1^CTD^-L anisosomes, showed a small but significantly lower t-half value and a larger mobile fraction estimated from the recovery curves of RIF1^CTD^-S and RIF1^CTD^-L anisosomes (Fig. 7, *I* and *J*). Due to the larger heterogeneity within RIF1^CTD^-L anisosome population (Fig. 7*H*) and the difficulty in measuring anisosome morphological recovery based on average fluorescent intensity, we also tabulated the frame numbers in which a bleached anisosome formed either a solid ball intermediate or a fully recovered “donut” as indicated in Figure 7*F*. The formation of both structures after photobleaching was significantly delayed for GFP-RIF1^CTD^-L *versus* GFP-RIF1^CTD^-S, suggesting that GFP-RIF1^CTD^-L anisosomes are intrinsically more stable than GFP-RIF1^CTD^-S anisosomes (Fig. 7, *K* and *L*). Although the reason for GFP-RIF1^CTD^-L anisosome recovery heterogeneity is unclear, it is conceivable that the expanded interactome for RIF1^CTD^-L or posttranslational modification of the S/K cassette influences its phase separation dynamics.

To investigate the potential role of phosphorylation in RIF1 CTD phase separation, we transfected U-2 OS cells with GFP-RIF1^CTD^-L and GFP-RIF1^CTD^-S CTDs harboring the corresponding 7SA/SD and 5SA/SD mutations (Fig. 6*A*). In contrast to the well-demarcated anisosomes formed by wildtype GFP-RIF1^CTD^-S, GFP-RIF1^CTD^-S^5SA^ formed irregularly shaped anisosomes that typically featured a narrow central cavity and thick outer shell. Loss of anisosomal character was even more pronounced for RIF1^CTD^-S^5SD^, which exclusively formed irregular nuclear aggregates (Fig. 7*M*). While the corresponding GFP-RIF1^CTD^-L^7SA^ and GFP- RIF1^CTD^-L^7SD^ mutants also exhibited anisosome morphology defects; the changes were less dramatic than those seen in GFP-RIF1^CTD^-S^5SA^ and GFP-RIF1^CTD^-S^5SD^. This was most pronounced for the SD mutations that completely disrupted GFP-RIF1^CTD^-S anisosomes but only partially inhibited GFP-RIF1^CTD^-L anisosome formation. The fact that the presence of S/K cassette reduced the impact of disruptive serine mutations on anisosome formation supports the conclusion that this motif stabilized phase separation of the RIF1 CTD.

## Discussion

RIF1 fulfills evolutionarily conserved and remarkably diverse roles in maintaining genome stability. Here, we show that DNA damage- and cell cycle-regulated alternative splicing contributes to RIF1 functional diversity by altering the biochemical properties of the RIF1 CTD. Consequently, RIF1-L and RIF1-S exhibit measurable differences in chromatin association, phase separation, and association with DNA repair factors. We propose that these functional differences allow for optimized RIF1 function in response to genotoxic stress and in different cellular contexts.

The alternative splicing of *RIF1*-Ex32 in mammalian cells was regulated by no less than six RNA binding proteins, including two enhancers (SRSF1 and snRNP70) and four inhibitors (PTBP1, SRSF3, SRSF7, and RBM28) which functionally interact with one another and may act cumulatively or synergistically to regulate Ex32 splicing. The central role for SRSF1 as an enhancer of Ex32 inclusion was supported by its association with RIF1 pre-mRNA, which was reduced in *RIF1-L*^*−/−*^ cells that harbor a point insertion (denoted by ˆ) in a CCCAGGAˆT SRSF1 recognition motif (Fig. 3*A* and 4*F*). *RIF1*-Ex32 was also identified as an SRSF1-regulated cassette exon in developing mouse epithelia by Yu *et al.* (34). Although snRNP70 binding was not detected (possibly due to poor antibody performance in RNA-IP), it is often found within the same alternative splicing complex as SRSF1 where it enhances recognition of the 5′ splice site (34, 79). Cooperative activities of SRSF1 and snRNP70 may promote *RIF1*-Ex32 inclusion in actively dividing progenitors and S/G_2_-phase cells where RIF1-L levels are high (Fig. 1, *I* and *J*).

DNA damage promoted Ex32 skipping, leading to increased production of RIF1-S mRNA and protein (Fig. 1). Inhibition of Ex32 splice-in could be caused by inhibition of splicing enhancer (*e.g.,* SRSF1), activation of splicing inhibitors (*e.g.,* SRSF3, SRSF7, and PTBP1), or a combination of mechanisms. Interestingly, while SRSF1, SRSF3, and SRSF7 were all induced by DNA damage (Fig. 4, *L* and *M*), only SRSF3 and SRSF7 showed increased occupancy over RIF1 transcripts in CLM-treated cells (Fig. 4, *I*–*K*). This suggests that the inhibitory effects of SRSF3 and SRSF7 binding are dominant to the positive effects of SRSF1 binding as it pertains to Ex32 inclusion in the setting of DNA damage. Because PS2 (Fig. 4*C*) detected a modest reduction in SRSF1-binding in CLM-treated cells (Fig. 4*I*), it is possible that SRSF3 and SRSF7 directly compete with SRSF1 for binding to sequences in the 3′ end of Ex32, which is consistent with the SRSF1 binding motif identified in our *RIF1-L*^*−/−*^ cells (highlighted in Fig. 3*A*). Perhaps relevant to these data, previous work has shown that SRSF3 levels are maintained within a narrow range through negative autoregulation, as well as positively regulated by SRSF1 and SRSF2 as reviewed in Ref. (40, 41, 42, 80). Hence, increased SRSF1 expression following DNA damage could activate SRSF3, thereby contributing to the RIF1-L to RIF1-S isoform switch. However, we noted that overexpression of SRSF1 alone was not sufficient to activate SRSF3, whereas attempts to overexpress SRSF3 activated its own negative autoregulation (Fig. 4, *N* and *O*). On the other hand, PTBP1 impairs SRSF3 autoregulation which likely explains the overexpression of both in primary cancers which predominantly express RIF1-S (34, 79, 80, 81) (Figs. 2 and S5*B*). It is perhaps noteworthy that the 3′ end of RIF1-In31 contains a conserved pyrimidine-rich tract (TTTTTTTCTCTCCTTTCTTCT) that may mediate PTBP1 binding. Regardless of the exact mechanism, our findings suggest that the rates of *RIF1*-Ex32 inclusion are determined by the competitive balance between splicing enhancers (*e.g.,* SRSF1) and inhibitors (SRSF3, SRSF7, and PTBP1), whose abundance and activities are regulated by DNA damage and the cell cycle. In the G_1_ phase and in the scenario of prolonged DNA damage, the inhibitory splicing factors prevail, leading to increased levels of RIF1-S. Deciphering potential cooperative interactions between these factors and understanding how their activities are affected by DNA damage will be the subject of future studies.

RIF1 chromatin proteomics identified both known and novel RIF1 interactants and pointed toward MDC1 as a potential target of regulation by RIF1-L (Fig. 5, *E*–*I*). Because MDC1 is also a chromatin-associated protein (82), its increased abundance in RIF1-L IPs could be due to the enhanced chromatin association of RIF1-L *versus* RIF1-S (Fig. 6, *F* and *G*). Alternatively, the S/K cassette could mediate direct interaction with MDC1. While the functional significance of the enhanced RIF1-L-MDC1 interaction is not fully understood, our findings suggest that RIF1-L sustains MDC1 accumulation at IR-induced foci and phosphorylation of its CTD reduces this property (Figs. 5, *H* and *I* and 6, *M* and *N*). This finding was somewhat unexpected given that RIF1 is recruited to IR-induced foci downstream of MDC1 through direct interaction with phosphorylated 53BP1 (28). How RIF1 promotes MDC1 focus formation is unclear. One speculation is that oligomerization of chromatin-bound RIF1-L participates in the recently described clustering of topologically associating domains that have incurred DNA damage (83), leading to local enrichment of MDC1. We also note that RIF1-L was reported to enhance 53BP1 nuclear body assembly in G_1_-phase cells (84). Although not tested here, such a function is conceptually consistent with the enhancement of DNA damage focus formation seen in our studies and might be akin to RIF1 function in the organization of topologically associating domains with shared replication timing (25). Further experiments are needed to establish mechanisms and functional consequences of the RIF1-L-MDC1 interaction.

While the functional significance is not known, the only protein that showed enhanced interaction with RIF1-S was the auxiliary NHEJ factor, PAXX. RIF1-S association with PAXX suggests the intriguing possibility that, in addition to its roles in suppressing HDR, RIF1 may also contribute an isoform-specific role in mediating NHEJ directly (Fig. 5*E*). Although this hypothesis remains to be tested, RIF1-L and RIF1-S U-2 OS cells exhibited distinct DSB repair profiles post-IR in neutral comet assays that may be compatible with differences in repair rate. Specifically, even though RIF1-S expressing cells were more susceptible to IR-induced DSBs, the breaks were repaired significantly faster compared to *RIF1*^*−/−*^ and RIF1-L expressing cells (Fig. 5, *J* and *K*). Although the precise nature of the RIF1-S–PAXX interaction awaits further study, we note that RIF1-S mRNA is induced by DNA damage and most abundant in G_1_ phase when NHEJ is the predominant DSB repair pathway (Fig. 1, *I* and *J*). NHEJ and HDR reporter assays using nonchromatinized substrates showed significant reduction in both the NHEJ and HDR efficiencies between wildtype and *RIF1-L*^*−/−*^ HEK 293T cells, likely due to the defect in sustained MDC1 recruitment to DSBs and a secondary consequence of reduced cycling rate. The study of cells selectively defective for RIF1-S should further inform its isoform-specific contributions to NHEJ.

The basic S/K cassette strengthened the chromatin binding of RIF1-L which was diminished through multisite phosphorylation of the CTD—including at least two sites within the S/K cassette—in response to WEE1i (Fig. 6, *D*–*L*). Previous work showed that WEE1 inhibitors induced CDK1-dependent phosphorylation of RIF1 on S2205, leading to PP1 dissociation, MCM4 hyperphosphorylation, and activation of dormant replication origins (73). Given that phosphorylation of S2260/65 and flanking CDK1 site occurs concomitant with S2205 phosphorylation (Fig. 6, *D* and *E*), we propose that chromatin eviction of RIF1 contributes to the loss of origin suppression seen in cells treated with WEE1 inhibitors (73).

The RIF1 CTD undergoes LLPS *in vitro* and phase-separates into anisosome-like structures in intact cells (78) (Fig. 7*B*). While both GFP-RIF1^CTD^-S and GFP-RIF1^CTD^-L formed anisosomes, they exhibited distinct characteristics. GFP-RIF1^CTD^-S anisosomes were larger, more dynamic, fusion prone (Fig. 7*C*) and exhibited faster FRAP recovery times with larger mobile fraction as a more homogenous species compared to GFP-RIF1^CTD^-L anisosomes (Fig. 7, *F*–*L*). GFP-RIF-L^CTD^ was less susceptible to disruption by phosphomimetic amino acid substitutions (Fig. 7*M*), which are well known to disrupt phase separation of fused in sarcoma (FUS) and other proteins that undergo LLPS (25, 85). This finding is consistent with studies showing that Ser-rich motifs can function as spacers to promote phase separation and droplet hardening (86). While full-length RIF1 also forms nuclear assemblies that may be attributed to phase-separation (Fig. 7*D*), it did not form discrete anisosomes, which may require supraphysiologic levels of RIF1 or may be suppressed by intramolecular folding. Nevertheless, stabilization of RIF1 LLPS by the S/K cassette may contribute to RIF1-dependent replication timing regulation, potentiation of MDC1 accumulation at IR-induced foci, 53BP1 nuclear body formation (84), and other chromatin-associated roles of RIF1.

While this article was under revision, Dong *et al.* reported that phosphorylation of S2265 in the RIF1-L S/K cassette mediated an interaction with the BRCT repeat of BRCA1 to facilitate HDR in response to replication stress (87). Our results provided a contradictory insight. HU treatment slightly increased RIF1-L/RIF1-S mRNA ratio (Fig. S1, *I* and *J*) which is likely due to the cell cycle arrest at S/G_2_ phase in which RIF1-L expression predominates (Fig. 1, *K* and *L*). Furthermore, the phosphorylation stoichiometry of the S2265 residue obligatory for BRCT domain binding was very low outside of mitosis and was not significantly induced by HU (Figs. 6*C* and S7, *A* and *B*). While AlphaFold3 also predicted an interaction between phospho-S2265 and the BRCT repeat of MDC1 that could theoretically promote the association between RIF1 and MDC1 during mitosis (Fig. S7*C*), MDC1 and RIF1 exhibited nonoverlapping localization patterns in mitotic cells (Fig. S8) where MDC1 remains functionally active (88). While functional implications of RIF1 phosphorylation remain to be fully elucidated, cophosphorylation of S2260, S2265, and other Ser residues distributed throughout the RIF1 CTD is consistent with an inhibitory mass charge effect on chromatin binding that has also been described for 53BP1 (89). Studies using cells from *RIF1-L*^*−/−*^ mice should further illuminate the roles of MDC1 and BRCA1 as RIF1-L effector proteins.

The functional differences between RIF1-L and RIF1-S described here may be relevant to RIF1 isoform usage differences between normal and cancer cells. We note that SRSF1 was upregulated in osteosarcoma cell lines, such as U-2 OS (44), that expresses high levels of RIF1-L (Figs. 1*C* and S5, *C*–*F*). In addition, the RIF1-L/RIF1-S isoform ratio was consistently reduced in primary cancers of diverse origin (Fig. 2). Whether or not this isoform switch drives aspects of tumorigenesis is unclear; however, reduced expression of RIF1-L may negatively impact MDC1-mediated signaling and other chromatin-associated roles for RIF1 that may suppress neoplastic growth. Nonexclusively, RIF1-S may function as a progrowth isoform and/or enhance DSB repair through interaction with PAXX or other factors. Further delineation of RIF1 isoform-specific functions *in vivo* will illuminate these possibilities in carcinogenesis.

## Experimental procedures

### Cell culture and treatment

U-2 OS, HeLa, H460, and HEK293T cell lines were obtained from the American Type Culture Collection. U-2 OS and its derivative cell lines were grown in McCoy’s 5A medium (Corning, 10-050-CV). HEK293T and HeLa cells were grown in Dulbecco's modified Eagle's medium (Corning, 10-013-CV). All cell lines were grown in medium supplemented with 10% fetal bovine serum (GeminiBio 900-108-500) and 1% penicillin/streptomycin (Corning, 30-002-CI) and incubated at 37 °C in 5% CO_2_. For G_1_/S synchronization experiments, cells were treated with 2 mM thymidine for 19 h, released into thymidine-free growth media for 9 h, and then returned to thymidine-containing media for an additional 16 h (90). The cells were washed three times with phosphate-buffered saline (PBS) and then released into complete media for the indicated time periods. CLM was prepared at a concentration of 4 μM stock solution in dimethyl sulfoxide (DMSO) and used at a concentration of 2 to 10 ng/ml for 4 to 6 h. For combinatorial treatments with CLM and DNA repair inhibitors, HeLa cells were pretreated with the indicated inhibitor (ATMi/KU-55933 at 10 μM; DNA-PKi/NU7741 at 2.5 μM; or PARPi/PJ-34 at 10 μM) for an hour before the 4 h treatment with 10 nM CLM. For checkpoint inhibitor treatments, U-2 OS cells were treated with 5 μM AZD6738 (ATRi) or 0.5 μM AZD1775 (WEE1i) diluted in DMSO for 1, 2, or 4 h prior to harvesting. To induce replication stress, HeLa cells were treated with 10 μM CPT or 50 μM DRB for 4 h. HU was prepared at a concentration of 1 M and added to the cells at the working concentration (2-5 mM) for the indicated time points (1, 2, 4, or 16 h).

Overexpression of SRSF1 and SRSF3 in HeLa cells was achieved by lipofectamine-mediated transient transfection (Thermo Fisher, L3000015) of pcDNA3.1-FLAG-SF2 (Addgene Plasmid #99021) or pcDNA3.2 V5-DEST 3XFlag-SRSF3 (Addgene Plasmid #46736) according to the manufacturer’s protocol.

### RNA extraction, *RIF1* splicing assay, and quantitative PCR

Total RNA was extracted in TRIzol reagent (Invitrogen, 15596018) followed by cDNA synthesis by iScript cDNA Synthesis Kit (Bio-Rad, 1708891) or iScript gDNA Clear cDNA Synthesis Kit (Bio-Rad, 1725035) according to manufacturer’s protocols. Human *RIF1*-Ex32 splicing was evaluated by RT-PCR using primers located on Ex31 and Ex33 (RIF1-Ex31-F: 5′-AAGCAGGATTGGCAGATGAC-3′ and RIF1-Ex33-R: 5′-GATGTCAACTGGTGCCACAC-3′). Positionally analogous primers located within mouse *Rif1*-Ex31 (5′-AAGCAGGATTGGCAGATGAC-3′) and Ex33 (5′-GATGTCAACTGATGCTGCAC-3) were used to analyze mouse *Rif1*-Ex32 splicing. Primers flanking Ex1a (RIF1-Ex1a-F: 5′-CGCCATCTTGGTCTAGGAGG-3′ and RIF1-Ex1a-R: 5′-ACGACTGGTCAGAGTCAGGT-3′) were used as a negative control. *SRSF3*-Ex4 splicing was assessed by primers located on Ex3 and Ex6 (SRSF3-Ex3-F: 5′-CGTCGCCCTCGAGATGATTA-3′ and SRSF3-Ex6-R: 5′-TCGGGACGGCTTGTGATTTC-3′). Beta-actin (Actin-F: 5′-TCCCTGGAGAAGAGCTACG-3′ and Actin-R: 5′-GTAGTTTCGTGGATGCCACA-3′) or glyceraldehyde-3-phosphate dehydrogenase (GAPDH) (GAPDH-F: 5′-AATCCCATCACCATCTTCCA-3′ and GAPDH-R: 5′-TGGACTCCACGACGTACTCA-3′) was used as an internal control. *RIF1* alternative splicing leads to the formation of RIF1-L and RIF1-S transcripts with a length difference of 78 bp which can be resolved subsequently by 2% w/v agarose gel electrophoresis (See Fig. 1*B*). Alternative splicing of *SRSF3*-Ex4 inclusion increases the transcript size by 456 bp which can be visualized by 1 to 2% agarose gel.

RIF1 isoform–specific qPCR in human cells was performed using the following primers: RIF1-L (5′-GGATTGGCAGATGACATTGATAGA-3’; 5-TCCTTTGGCTGAAGTGGTATTATG-3′); RIF1-S (5′-CCTACTACACAATCTAAGATTTCA-3′; 5′-GCTCTAATGAGTTGTCCCA-3′); and total RIF1 (5′-CGCTGTGTCTGGTCTCCTT-3′; 5′GCACCGTCTATCAATGTCATCTG-3′) with iTaq Universal SYBR Green Supermix (Bio-Rad, 1725124) according to manufacturer’s protocols.

### Gene editing and cloning

*RIF1*^*−/−*^ and *RIF1*^*ΔEx32*^ cells were generated by transient transfection of U-2 OS cells with pX459 vectors (v2, Addgene plasmid #62988) (91, 92) harboring two single guide RNA (sgRNA) sequences targeting *RIF1*-Ex2 (5′-CACCgAGTCTCCAACAGCGGCGCGA-3′ and 5′- AAACTCGCGCCGCTGTTGGAGACTc-3′) or *RIF1*-Ex32 (5′-CACCgATTTAGGGCTACGTGATCCT-3′ and 5′-AAACAGGATCACGTAGCCCTAAATc-3′) using jetPRIME (Sartorius, 101000046). Twenty-four hours after transfection, cells were selected for 72 h with 1 μg/ml puromycin and then diluted into 96-well plates at an average density of 1 cell per well. Each single clone was isolated and screened for RIF1 knockout phenotype through immunostaining of ionizing radiation-induced foci and Western blotting with α-RIF1 and α-RIF1-L antibodies. All clones were sequenced around the sgRNA-targeted sequence, and five clones (*RIF1*^*−/−*^: H1 and 2C5; *RIF1-L*^*−/−*^: A6, 2A2, and H11) were selected for further study.

We reconstituted *RIF1*^*−/−*^ U-2 OS cells with full-length RIF1-L and RIF1-S coding sequences cloned into a tetracycline-inducible pcDNA5-eGFP-FRT/TO plasmid vector (Addgene plasmid #19444) by Gateway recombination cloning (Invitrogen, 11789020 and 11791020) (6). Resulting GFP-RIF1-L and GFP-RIF1-S plasmids were cotransfected into *RIF1*^*−/−*^ Tet-on U-2 OS cells with pOG44 using jetPRIME, selected with 200 μg/ml hygromycin for 1 week, and tested for RIF1 expression following induction with 1 μg/ml Dox. GFP plasmid was included as a control. The three resulting cell lines *RIF1*^*−/−*^:GFP, *RIF1*^*−/−*^:GFP-RIF1-L, and *RIF1*^*−/−*^:GFP-RIF1-S are also being referred as GFP, GFP-RIF1-L, and GFP-RIF1-S expressing cells throughout this paper. *RIF1*^*−/−*^ U-2 OS cells expressing GFP-tagged RIF1^CTD^ constructs were generated by PCR amplifying codons 2170 to 2472 of the RIF1-L or RIF1-S coding sequences followed by Gateway cloning into pcDNA5-eGFP-FRT/TO. Point mutations were introduced through QuikChange mutagenesis method with Phusion High Fidelity DNA Polymerase (Thermo Scientific, F530) or KOD Hot Start Polymerase (Novagen, 71086). All constructs were sequenced in their entirety. GFP-RIF1-L^CTD^ and GFP-RIF1-S^CTD^ constructs were stably transfected into *RIF1*^*−/−*^ U-2 OS cells as described above. Experimentally verified CDK1 phosphorylation sites of human RIF1 protein were queried from UniProt website under the primary accession ID: Q5UIP0 on 6th July 2022.

### Generation of RIF1-L-deficient mice

One-cell embryos from C57BL/6J mice were injected with a mixture of 40 ng/μl of Cas9 protein (PNA Bio) and 25 ng/μl of each of the two sgRNAs complementary to the 3′ portion of *Rif1*-In31 (sgRNA1: CCTAACATTTTACAAGGGCGATT) and 5′ portion of *Rif1*-Ex32 (sgRNA2: CCCAGGATCACAGAGCTCTAAAT) of the m*Rif1* gene spanning nucleotides 51962725 to 52016781 of mouse chromosome 2 (Reference GRCm39 C57BL/6J). The region flanked by sgRNA1 and sgRNA2 comprised of 149 bp sequence that includes *Rif1*-Ex32 5′ splice acceptor site. Tail DNA from founder mice was subjected to deep sequencing using m*Rif1* primers, and those mice exhibiting *Rif1* mutations were backcrossed to wildtype mice to obtain germline m*Rif1* mutant lines. Two lines were selected to be used in this study: a *Rif1*^*iA*^ line harboring a single nucleotide (A) insertion at codon 2010 that likely reflects cleavage and error-prone repair at sgRNA2, and a *Rif1*^*ΔEx32*^ line harboring a 129 bp deletion between sgRNA1 and sgRNA2 that deletes the 3′portion of In31and the 5′ portion of Ex32, including the 5′ splice site. Analysis of T and B cell development, mitogenesis, and *in vitro* class switch recombination were carried out using cells isolated from bone marrow and spleen of 6-to-8-week old *Rif1*^*+/+*^ and *Rif1*^*ΔEx32/ΔEx32*^ mice as previously described (93, 94). All murine studies were approved by IACUC – RARC University of Wisconsin-Madison (M005156-R03).

### RNAi and shRNA screening

siRNA screen for *RIF1* splicing regulators was performed through a SMARTpool siRNA library (Dharmacon) consisting of 144 siRNAs targeting RNA-binding proteins in the human genome that was obtained through UW Small Molecule Screening & Synthesis Facility (95). Approximately, 10,000 HeLa cells were reverse-transfected with 20 nM of each of the siRNAs using DharmaFECT1 (Dharmacon) in 96-wells plates for a 48-h period (Fig. S4*A*). Subsequently, the transfected cells were lysed in TRIzol reagent for RNA extraction, and cDNA was synthesized as described. *RIF1* splicing assay was performed as described to identify candidate *RIF1* splicing factors.

The following shRNA lentiviral vectors targeting candidate *RIF1* splicing regulators were purchased from Sigma: SRSF1 (cat# TRCN0000001095); SRSF2 (cat# TRCN0000000084); SRSF3 (cat# TRCN0000001227); SRSF7 (cat# TRCN0000001142); PTBP1 (cat# TRCN0000231420); RBM28 (cat# TRCN0000239461); and snRNP70 (cat# TRCN0000000011). Nontargeting vector (Addgene plasmid #1864) was included as negative control. Lentiviral particles were produced by transient transfection of HEK293T cells with shRNA vectors, psPAX2 (Addgene plasmid #12260), and pCMV-VSV-G (Addgene plasmid #8454) in a ratio of 4:3:3 by jetPRIME as described (90, 96). Viral supernatants harvested at 24 h and 48 h posttransfection were incubated with U-2 OS cells for 24 h followed by selection in media containing 2 μg/ml puromycin for 48 to 72 h. Cells were harvested in TRIzol reagent for *RIF1* splicing assays.

### RNA immunoprecipitation

Approximately 50 million cells were lysed in 1 ml ice-cold NET-2 buffer [50 mM Tris-HCl (pH 7.5), 150 mM NaCl, 0.05% v/v NP40] supplemented with 2 mM 1,4-dithiothreitol, 0.2 U/μl RNasin Plus (Promega, N2611), 20 mM sodium fluoride, 20 mM β-glycerophosphate, and 2X protease inhibitor cocktail (Sigma, P8340-5ml; Thermo Scientific, 78438). The lysate was sonicated using five pulses of 3 s ON, 30 s OFF followed by three pulses of 10 s ON and 30 s OFF at an amplitude of 30% (Fisher Scientific, FB120). The lysate was cleared by centrifugation at 14,000*g* for 10 min, 4 °C. The supernatant was incubated with 5 μg of either the targeted antibodies (SRSF3: Cell Signaling Technology #35073; SRSF7: Bethyl A303-772A; PTBP1: Thermo Fisher 32-4800; SRSF1-M: Santa Cruz sc33652; SRSF1-R: Abcam ab38014; SRSF2: Proteintech 20371-1-AP; snRNP70: Invitrogen #PA5-115943) or normal IgG controls (Mouse: Millipore 12-371; Rabbit: Millipore 12-370) for 1 h on a nutator mixer at 4 °C before Protein A/G PLUS-Agarose bead suspension (Santa Cruz, sc-2003) was added (20 μl/1 μg of antibody) for overnight incubation. The beads were washed with NET-2 buffer five times, and the immunoprecipitated RNA was extracted by TRIzol reagent. Relative log_2_ fold enrichment of RIF1 pre-mRNA in the target RNA-IP sample *versus* the control IgG sample or the percent input was quantified by qPCR assay with two pairs of intron-exon primers. Primer set 1 targets *RIF1*-In31 and the 5′ end of *RIF1*-Ex32 while PS2 targets the 3′ end of *RIF1*-Ex32 and *RIF1*-In32 (RIF1-In31-F: 5′-TAGTCATCTAGGGTTCTGAGTG-3′ and RIF1-Ex32-R: 5′-TCCTTTGGCTGAAGTGGTATTATG-3′; RIF1-Ex32-F: 5′-CATAATACCACTTCAGCCAAAGG-3′ and RIF1-In32-R: 5′- GTGACATGAAAACTAAAGCACTTC-3′).

### DNA replication pattern analysis

DNA replication pattern analyses were performed as described (90). *RIF1*^*−/−*^, *RIF1*^*−/−*^:GFP, *RIF1*^*−/−*^:GFP-RIF1-L, and *RIF1*^*−/−*^:GFP-RIF1-S U-2 OS cells were pulse labeled with 20 μM EdU for 20 min and stained for EdU incorporation. The presence of early, mid, or late DNA replicative stages were accessed from the EdU staining patterns. The percentages were calculated for each sample. Alternatively, cells were synchronized with 2 mM thymidine for 19 h, released into thymidine-free growth media for 9 h, and then returned to thymidine-containing media for 6 h, at which time most cells are in mid-S phase. Cells were then pulse labeled with EdU as described. A minimum of 100 cells per sample was imaged by confocal microscopy.

### EdU labeling, flow cytometry, immunofluorescent microscopy, and live cell imaging

For cell cycle progression experiments, U-2 OS cells were incubated with 20 μM EdU for 30 min before collection and then fixed with ice-cold 70% ethanol. EdU detection was performed using the Click-IT Plus EdU Alexa Fluor 647 Flow Cytometry Assay Kit (Life Technologies, C10634). Propidium iodide was added at a concentration of 50 μg/ml. Flow cytometry was performed on Thermo Fisher Attune, and data were analyzed and organized using FlowJo software. For *in situ* EdU and 5-bromo-2′-deoxyuridine (BrdU) staining, U-2 OS cells were pulse labeled with 20 μM BrdU or EdU for 30 min and fixed with 4% w/v paraformaldehyde. For BrdU detection, cells were then incubated with 2 M HCl for 30 min and then permeabilized with 0.2% v/v Triton X-100 for 15 min at room temperature, washed, and blocked in 3% w/v bovine serum albumin (BSA). Cells were stained with BrdU primary antibody (Santa Cruz, sc-32323) in 3% BSA and incubated overnight at 4 °C, followed by washing in 0.02% v/v PBST (PBS with 0.02% v/v Tween-20) and incubation with appropriate secondary antibodies in 3% BSA for 1 h at room temperature. EdU was detected by click chemistry as described above. Samples were mounted in VECTASHIELD mounting medium with 4′,6-diamidino-2-phenylindole (DAPI) (Vector, H-1200) before imaging.

For immunostaining experiments, cells were seeded into 12-well plate with glass coverslips, fixed with 4% w/v paraformaldehyde, permeabilized with 0.2% v/v Triton X-100, and blocked with 3% w/v BSA at room temperature. The coverslips were then transferred to an improvized humidity chamber for immunostaining with the appropriate primary antibodies at 37 °C for an hour, room temperature for 2 h, or at 4 °C overnight. Primary antibodies and the dilution factors used were listed in figure legends. Coverslips were washed 3 × 10 min with 0.05% v/v PBST (PBS with 0.05% v/v Tween-20) before incubating with Alexa Fluor secondary antibodies (Thermo Fisher #A11032, #A32733) at a dilution factor of 1:10,000 at room temperature for 45 min. Coverslips were washed 3 × 10 min with 0.05% PBST followed by 2 × 1 min with PBS before mounting. Nuclear DNA was either stained with 0.5 μg/ml DAPI for 10 min at room temperature and then mounted with mounting medium for fluorescence (Vector, H-1000) or directly mounted in mounting medium with DAPI for fluorescence (Vector, H-1200) before imaging. Images were acquired using Nikon A1RS or Nikon AX confocal microscopes under the desired objectives with Airy Unit = 1. Images were organized using NIS-Elements Advanced Research v5.20.02/Fiji ImageJ software. Foci counts were done in CellProfiler (v4.2.6) or ImageJ macro Foci_Analyzer_1_5 (https://imagej.net/plugins/foci-analyzer). Anisosome area measurement was performed in Fiji utilizing Labkit classifier followed by manual annotation. Outputs were extracted and visualized in R, MATLAB (R2023a), and/or Prism (v10, GraphPad).

For live cell imaging, cells were plated into 38 mm glass bottom dishes and stained with 1 μg/ml of Hoechst 33342 for 30 min before being imaged in humidified CO_2_ chamber supplemented with 5% CO_2_ throughout the experiment.

### *In vitro* DNA binding assay

Fluorescein-labeled antiparallel G4 DNA (5′-FAM-TTT TTT GGG GGG GGG GGG GGG GGG GG-3′) was folded in refolding buffer [1 μM DNA, 10 mM Tris-HCl (pH 7.5), 50 mM KCl, 1 mM ethylenediaminetetraacetic acid (EDTA), 40% v/v polyethylene glycol 200] by heating to 95 °C for 10 min and subsequent cooling over 4 h. Purified RIF1 CTD was incubated with 5 nM DNA in reaction buffer [20 mM Hepes (pH 7.6), 50 mM KCl, 1 mM EDTA, 0.01% v/v Triton X-100, 10% v/v polyethylene glycol 200] for 30 min at 30 °C. The fluorescence anisotropy of each sample was measured at 25 °C with a Beacon 2000 fluorescence polarization system.

### Protein extraction, chromatin fractionation, and immunoblotting

For whole-cell protein extraction, cells were lysed in either high salt lysis buffer [50 mM Tris (pH 7.5), 300 mM NaCl, 10% glycerol, 2 mM MgCl_2_, 3 mM EDTA, and 0.5% Triton X-100] or modified radioimmunoprecipitation assay (RIPA) buffer [50 mM Tris-HCl (pH 7.4), 150 mM NaCl, 1 mM EDTA, pH 8, 1% sodium deoxycholate, 0.1% SDS, and 1% Triton X-100] supplemented with 10 mM sodium fluoride, 10 mM β-glycerophosphate, and 1X Protease Inhibitor Cocktail (Sigma, P8340-5ml; Thermo Scientific, 78438). The lysate was incubated on ice for 20 min and sonicated using five pulses of 3 s ON, 5 s OFF at an amplitude of 30% (Fisher Scientific, FB120) before the addition of 4X SDS sample buffer [200 mM Tris-HCl (pH 6.8), 40% glycerol, 8% SDS, 0.5% bromophenol blue and 10% beta-mercaptoethanol]. The samples were heated at 95 °C for 5 min prior to freezing at −20 °C for storage or loading directly for immunoblotting.

For chromatin fractionation of full-length RIF1, cells were resuspended in CSK buffer [20 mM Hepes (pH 7.4), 150 mM NaCl, 3 mM MgCl_2_, 300 mM sucrose, and 0.5% Triton X-100] supplemented with 20 mM sodium fluoride, 20 mM β-glycerophosphate, and 2X protease inhibitor cocktail. The cells were incubated on ice for 20 min. Fifty percent of the cell suspension was kept as whole cell extract with the addition of 50 U/ml benzonase (Sigma, E1014-5KU) followed by a 20 min on-ice digestion. The remaining cell suspension was centrifuged for 5 min at 5000*g* at 4 °C. The supernatant was transferred to a new tube and saved as the SF which contains cytoplasmic and nucleoplasmic content, while the pellet/chromatin fraction was washed twice in CSK buffer without detergent and resuspended in complete CSK buffer with 50 U/ml benzonase for a 20 min on-ice digestion. Each 100 μl of lysates were mixed with 50 μl of 4X SDS sample buffer and heated at 95 °C for 15 min before loading. Chromatin fractionation of the RIF1 CTD constructs was done with the same procedure, but the salt concentration of the CSK buffer was reduced to 100 mM due to the higher solubility of RIF1 CTD compared to full-length RIF1.

For immunoblotting, samples were separated by 6%, 12%, or 15% SDS-polyacrylamide gel depending on the molecular weight of the target protein and transferred to 0.45 μm Immobilon-FL PVDF membranes (MilliporeSigma, IPFL00010). For small molecular weight RBP transfer, 0.2 μm Amersham Protran Western blotting nitrocellulose membranes (MilliporeSigma, GE10600001) was used in Tris-glycine transfer buffer supplemented with 10% v/v methanol. For large molecular weight transfer, 0.01% v/v SDS was added in the transfer buffer. The membranes were blocked with blocking solution [5% w/v milk in Tris-buffered saline, 0.1% v/v Tween 20 (TBST)] for an hour before blotting with target primary antibodies overnight at 4 °C. The source and the dilution of the primary antibodies used were listed as followed: Total RIF1 (Bethyl Laboratories A300-569A; 1:500); GFP (Santa Cruz sc9996, 1:100); MCM2 (Santa Cruz sc373702, 1:100 or Abcam ab4461, 1:1000); MCM4 (Abcam ab4459, 1:1000); vinculin (Santa Cruz sc73614, 1:1000); SRSF1 (Abcam ab38014, 1:1000); SRSF2 (Proteintech 20371-1-AP, 1:1000); SRSF3 (Cell Signaling Technology #35073, 1:1000); SRSF7 (Bethyl A303-772A, 1:1000); PTBP1 (Thermo Fisher 32-4800, 1:1000); Phospho-histone H2A.X (Ser139)/gH2A.X (Cell Signaling Technology #9718S, 1:1000); CHK1 (Cell Signaling Technology #2360S, 1:1000); GAPDH (Cell Signaling Technology #2118S, 1:1000); lamin B1 (Abcam ab16048, 1:2000); α-tubulin (Sigma T6199, 1:1000); and β-tubulin (Sigma Millipore 05-661, 1:1000). After that, the membranes were washed 3 × 5 min with TBST and incubated with LI-COR IRDye secondary antibodies (IRDye 800CW goat anti-rabbit or IRDye 680RD goat anti-mouse) at a dilution of 1:10000 in blocking solution for an hour at room temperature. Membranes were washed 3 × 5 min with TBST, and images were acquired using Odyssey Fc/XF (LI-COR Biosciences). The exported images were then analyzed and organized with ImageStudio software (v5.2, LI-COR Biosciences).

### RIF1 isoform-specific antibodies

α-RIF1-L, α-RIF1-S, and α-RIF1-pS2260/65 antibodies (Lifetein, LLC) were peptide-affinity purified from the sera of rabbits injected with the following KLH-conjugated immunogens, respectively: hRIF1-S (N-VKTSPTTQSKISEMAKESIP-C); hRIF1-L (N-AKGFLSPGSRSPKFKSSKKC-C); and RIF1-pS2260/65 (N-AKGFL[pS]PGSRPKFKSSKKC-C). α-RIF1-pS2260/65 antisera were first immunodepleted against an identical nonphosphorylated peptide prior to affinity purification using the phosphopeptide. All antibodies were used at a dilution of 1:500 for Western blotting and immunofluorescence staining experiments.

### RIF1 purification and MS

RIME assay of GFP-RIF1-L and GFP-RIF1-S expressed on a *RIF1*^*−/−*^ background was carried out as described (46, 90) (Fig. 5*A*). Briefly, ∼20 million cells were counted and fixed with 20 ml 1% formaldehyde solution for 8 min at room temperature. Fixation was quenched by adding 0.12 M glycine. The SF was extracted in 10 ml of LB1 [50 mM Hepes-KOH (pH 7.5), 140 mM NaCl, 1 mM EDTA, 10% glycerol, 0.5% NP-40, 0.25% Triton X-100, and 1X Protease Inhibitor Cocktail (Sigma, P8340-5ml)] for 10 min with rotation at 4 °C. Cell nuclei were pelleted and washed once with 10 ml LB2 [10 mM Tris-HCl (pH 8.0), 100 mM NaCl, 1 mM EDTA, 0.5 mM ethylene glycol-bis(β-aminoethyl ether)-N,N,N′,N′-tetraacetic acid, 1X protease inhibitor cocktail] and then resuspended in 500 μl LB3 [10 mM Tris-HCl (pH 8.0), 100 mM NaCl, 2.5 mM MgCl_2_, 0.1% w/v sodium deoxycholate, 0.5% Triton X-100, 1X protease inhibitor cocktail] with 500 U benzonase and incubated at room temperature for 30 min. Benzonase was deactivated with 2 mM EDTA and 1 mM ethylene glycol-bis(β-aminoethyl ether)-N,N,N′,N′-tetraacetic acid. The mixture was supplemented with 50 μl 10% Triton X-100 and 37.5 μl of 4 M NaCl before LB3 was added to bring the total lysate volume of each sample to 1 ml. Digested lysates were sonicated for three pulses of 10 s ON, 50 s OFF at an amplitude of 40%, and clarified by centrifugation at 20,000*g* for 10 min at 4 °C. The supernatants were incubated with ChromoTek GFP-Trap Magnetic Agarose beads (Fisher Scientific) per manufacturer’s recommendations on a nutator mixer at 4 °C overnight. Subsequently, 50 μl of prewashed Dynabeads protein G (Invitrogen, 10003D) was added to the lysates and incubated for additional 4 h at 4 °C.

For Western blotting, beads were washed sequentially with 1 ml LB3 and 1 ml RIPA buffer [50 mM Hepes-KOH (pH 7.5), 0.5 M LiCl, 1 mM EDTA, 1% NP-40, 0.7% w/v sodium deoxycholate, 1X protease inhibitor cocktail] and boiled in 100 μl 2X SDS sample buffer. For mass spectrometry, beads were washed five times with 1 ml of RIPA buffer and twice in 1 ml of ice-cold freshly prepared 100 mM ammonium bicarbonate solution and processed as described (46). Briefly, GFP-RIF1 RIME IPs were subjected to in-solution tryptic digestion by the addition of 15 μl of 10 ng/μl trypsin (Promega) in 100 mM ammonium bicarbonate solution to the washed beads directly. Samples were digested overnight at 37 °C. After overnight digestion, an additional 10 μl of the same trypsin solution was added to each sample and incubated for an additional of 4 h at 37 °C. The tubes were then placed on a magnetic rack, and the supernatant (approx. 25 μl) containing the digested peptides was removed to a fresh tube.

The tryptic digest solution was diluted to 100 μl with a final concentration of 0.1% trifluoroacetic acid (TFA) and desalted/concentrated using an Omix 100 μl (80 μg capacity) C18 tip. The solution was pipetted over the C18 bed five times and rinsed three times with H_2_O and 0.1% TFA to desalt. The peptides were eluted from the C18 resin into 150 μl elution solution (70% acetonitrile and 0.1% TFA) and lyophilized. The peptides were resuspended in 95:5 H_2_O:acetonitrile and 0.2% formic acid and analyzed as described below.

Peptides from four biological replicates of each condition were separated and analyzed using a UPLC-ESI-MS/MS system consisting of a NanoAcquity ultra-high-pressure liquid chromatography system (Waters) and an Orbitrap Q Exactive HF mass spectrometer (Thermo Fisher Scientific). UPLC separation employed an in-house constructed 100 × 365 μm fused silica capillary microcolumn packed with 20 cm of 1.7 μm-diameter, 130 Å pore size, and C18 beads (Waters BEH), with an emitter tip pulled to approximately 1 μm using a laser puller (Sutter instruments). Peptides were loaded in buffer A (H_2_O, 0.2% formic acid) at a flow-rate of 400 nl/min for 30 min and eluted over 120 min at a flow rate of 300 nl/min with a gradient of 5% to 35% acetonitrile, in 0.1% formic acid. The nanocolumn was held at 60 °C using a column heater (in-house constructed). The nanospray source voltage was set to 2200V. Full-mass profile scans were performed in the orbitrap between 375 and 1500 *m/z* at a resolution of 120,000, followed by MS/MS HCD scans of the 10 highest intensity parent ions at 30% relative collision energy and 15,000 resolution, with a 2.5 m/z isolation window and a mass range starting at 100 *m/z*. Charge states 2 to 6 were included, and dynamic exclusion was enabled with a repeat count of one over a duration of 15 s.

### Bioinformatic analyses of RIF1 RIME

The MetaMorpheus software program (version 0.0.32) was used to identify peptides and proteins in the samples (97, 98). Protein fold changes were quantified for each of the four biological replicates by FlashLFQ with match between runs enabled (99, 100). The UniProt-reviewed human database (downloaded on 10/05/2023 12:29:34) was used for calibration, global posttranslational modification discovery, and search. Default parameters were used including a maximum of two missed cleavages and minimum peptide length of seven amino acids for a tryptic digest. Carbamidomethylation of cysteine was set as a fixed modification, while oxidation of methionine was a variable with two maximum modifications per peptide. Differential expression analysis of the quantified protein groups was performed in Perseus (version 1.6.15.0) (101). Decoy proteins were filtered out as well as protein groups above a protein Q-value threshold of 0.01. The first biological replicate of RIF1-L and the fourth replicate of RIF1-S were not included in subsequent analysis due to its low data quality. The quantified intensities were log_2_ transformed and normalized by subtracting the median intensity for each column. A minimum of 70% valid values was required before imputation. Imputation involved replacing missing values from a normal distribution with the default Perseus parameters. Two sample student *t*-tests were performed with truncation by permutation-based FDR of 0.05. ANOVA was performed with 0.05 permutation-based FDR truncation followed by a *post hoc* Tukey’s HSD test to determine the significant pairs.

### Proximity ligation assay

*RIF1*^*−/−*^ U-2 OS cells expressing GFP, GFP-RIF1-L, or GFP-RIF1-S were mock irradiated or exposed to 10 Gy IR followed by 2 h recovery. The cells were then pre-extracted for 8 min with CSK buffer containing 100 mM NaCl and 0.5% v/v Triton X-100 to reduce cytoplasmic background signal prior to cell fixation and primary antibodies incubation (α-GFP, Santa Cruz sc9996, 1:250; α-MDC1, Sigma HPA006915, 1:500) overnight. Reagent kits for Duolink Proximity Ligation Assay (Sigma) were used, and PLA was performed according to the manufacturers’ conditions. PLA foci number for each genotype was quantified by customized R scripts.

### Neutral comet assay

*RIF1*^*−/−*^ U-2 OS cells expressing GFP, GFP-RIF1-L, or GFP-RIF1-S were mock irradiated or exposed to 10 Gy IR and harvested immediately (T0) or allowed for an hour of recovery in 37 °C (T1). Neutral comet assay was performed according to Trevigen’s protocol. Cells were harvested in 1X PBS (Trevigen #4870-500) and combined with molten CometAssay LMAgarose (Trevigen #4250-050-02) at 37 °C at a ratio of 1:10 (v/v) to reach a final cell density of ∼1 x 10^5^/ml. For each sample, 50 μl of the cell suspension was added onto a well of the CometSlide (Trevigen #4250-050-03). The slides were allowed for gelling for 30 min in the dark at 4 °C before being incubated in Lysis Solution (Trevigen #4250-050-01) at 4 °C overnight. After the slides were equilibrated in 1X TBE buffer (90 mM Tris, 90 mM boric acid, 2 mM Na_2_EDTA, pH 8.33) for 15 min, the nucleoids were subjected to electrophoresis at 22 V, 30 min at 4 °C in the dark. The slides were then immersed in distilled water for 5 min followed by 5 min in absolute ethanol at room temperature for fixation. The slides were subsequently dried at 37 °C for 15 min and then kept at room temperature for storing. Prior to imaging, the nucleoids were stained with 10 μg/ml propidium iodide for 30 min at room temperature. The slides were immersed in distilled water briefly, dried on paper towel, and imaged immediately. Comet images were segmented by OpenComet (102) in Fiji using the default parameter. Inaccurate segmentations were removed manually. Tail DNA percent of each segmented comet was calculated by OpenComet and extracted from its output using a custom MATLAB (R2023a) script.

### NHEJ and HDR reporter assay

Reporter assays were performed to assess DNA repair efficiency as described (103, 104). Wildtype HEK293T, which predominantly expressed RIF1-L and two of its CRISPR/Cas9–generated *RIF1-L*^*−/−*^ clones, were seeded in 6-wells plate. Once the cells reached 50 to 60% confluency, cells were transfected with 1 μg of NHEJ (EJ5-GFP) or HDR (DR-GFP) reporter, 1 μg of mCherry, and 1 μg of I-Sce I-expressing plasmids in 5 μl P3000 and 7.5 μl Lipofectamine 3000 reagents (Thermo Fisher, L3000015), each diluted in 125 μl serum-free Dulbecco's modified Eagle's medium. The DNA-lipofectamine mixture was vortexed followed by incubation at room temperature for 15 min. Cells were changed with 1 ml of fresh medium, and the DNA–lipofectamine mixture was added onto the cells dropwise. Twenty-four hours posttransfection, the cells were changed with 2 ml of fresh medium and incubated for additional 48 h. The cells were harvested and washed once before being resuspending in PBS supplemented with 2% v/v fetal bovine serum for analysis on Thermo Fisher Attune N x T flow cytometer. Mock-transfected cells (I-Sce I-expressing plasmid only), GFP-positive cells (EJ5-GFP + I-Sce I-expressing plasmids), and mCherry-positive cells (mCherry + I-Sce I-expressing plasmids) were included for each cell line for voltage calibration of the flow cytometry and as experimental controls. Data were analyzed and organized using FlowJo (v10.10). The total percentage of GFP-positive cells were normalized to the transfection efficiency in the total percentage of mCherry-positive cells.

### *In vitro* phase separation assay

RIF1-L and RIF1-S CTD fragments were subcloned into pDEST15 for expression as GST fusion proteins. GST-tagged RIF1 CTD fragments were transformed into BL21-AI strain. Protein expression was induced by growing the transformed bacteria in TB medium supplemented with 1 mM isopropylthio-β-D-1-galactoside (IPTG) + 0.2% w/v arabinose and grown at 16 °C overnight. Cells were then lysed in lysis buffer [50 mM Hepes (pH 7.5), 300 mM NaCl, 1 mM 1,4-dithiothreitol, 100 mM dextrose, 10% glycerol, and 1X protease inhibitor cocktail] by sonication. The SFs were collected by centrifugation at 20,000*g* for 30 min at 4 °C. The proteins were purified with GS4B resin and eluted in GS4B elution buffer [20 mM Hepes (pH 7.4), 300 mM NaCl, 10% glycerol, 20 mM reduced glutathione, 200 mM trehalose]. Purified GST-RIF1^CTD^-L and GST-RIF1^CTD^-S were then subjected to phase separation assays in the presence of 10% dextran/polyethylene glycol as described (105). Images were collected at 10 min postmixing.

### Fluorescence recovery after photobleaching

U-2 OS cells were seeded in 38-mm glass bottom dishes and transfected at 60% to 80% confluency with 2.5 μg of GFP-RIF1^CTD^ plasmid using Lipofectamine 3000 (Thermo Fisher, L3000015) in 7.5 μl Lipofectamine and 5 μl P3000 reagents, each diluted in 125 μl serum-free McCoy’s 5A medium. The transfection medium was changed at 4 h posttransfection, and the cells were incubated overnight for transgene expression. Cells were kept in humidified CO_2_ chamber supplemented with 5% CO_2_ throughout the experiment. FRAP was programmed through ND Stimulation module (Nikon NIS-Elements AR) on Nikon AX confocal microscope 24 h posttransfection with 25% 488 nm laser for 1.02 s on anisosomes of roughly 2.5 nm in diameter. Recovery images were acquired at 5 s interval for 3 min postbleaching. The resulting videos were analyzed in MATLAB scripts (https://github.com/adenine-koo/FRAP.git) according to the easyFRAP pipeline (106). Briefly, the average pixel values of three regions of interest (nucleus, bleached anisosome, and background) were extracted. Frame stabilization of the bleached anisosome was performed based on a correlation algorithm. Full-scale normalization was done to correct background fluctuation, starting intensity difference, acquisition bleaching/laser fluctuation as well as variation in bleaching depth. Each recovery curve was plotted and fitted by a single exponential equation to estimate its t-half value and mobile fraction.

### Statistical processing

Statistical analysis information including individual biological and technical replicates number, mean or median, and error bars were explained in the figure legends. Statistical tests were performed in Prism (v10, GraphPad) or R. The tests performed were described in the figure legends, and the resulting *p* values were shown in the respective figure.

## Data availability

Raw data will be available upon request.

## Supporting information

This article contains supporting information (43).

## Conflict of interest

The authors declare that they have no conflicts of interest with the contents of this article.
