# Supplementary material for: DNA-damage dependent isoform switching modulates RIF1 DNA repair complex assembly and phase separation
Source: J Biol Chem. 2025 Oct 24;301(12):110857. doi: 10.1016/j.jbc.2025.110857 (PMC12702018; doi:10.1016/j.jbc.2025.110857)
Supplement: Supporting Figures [file mmc1.pdf]

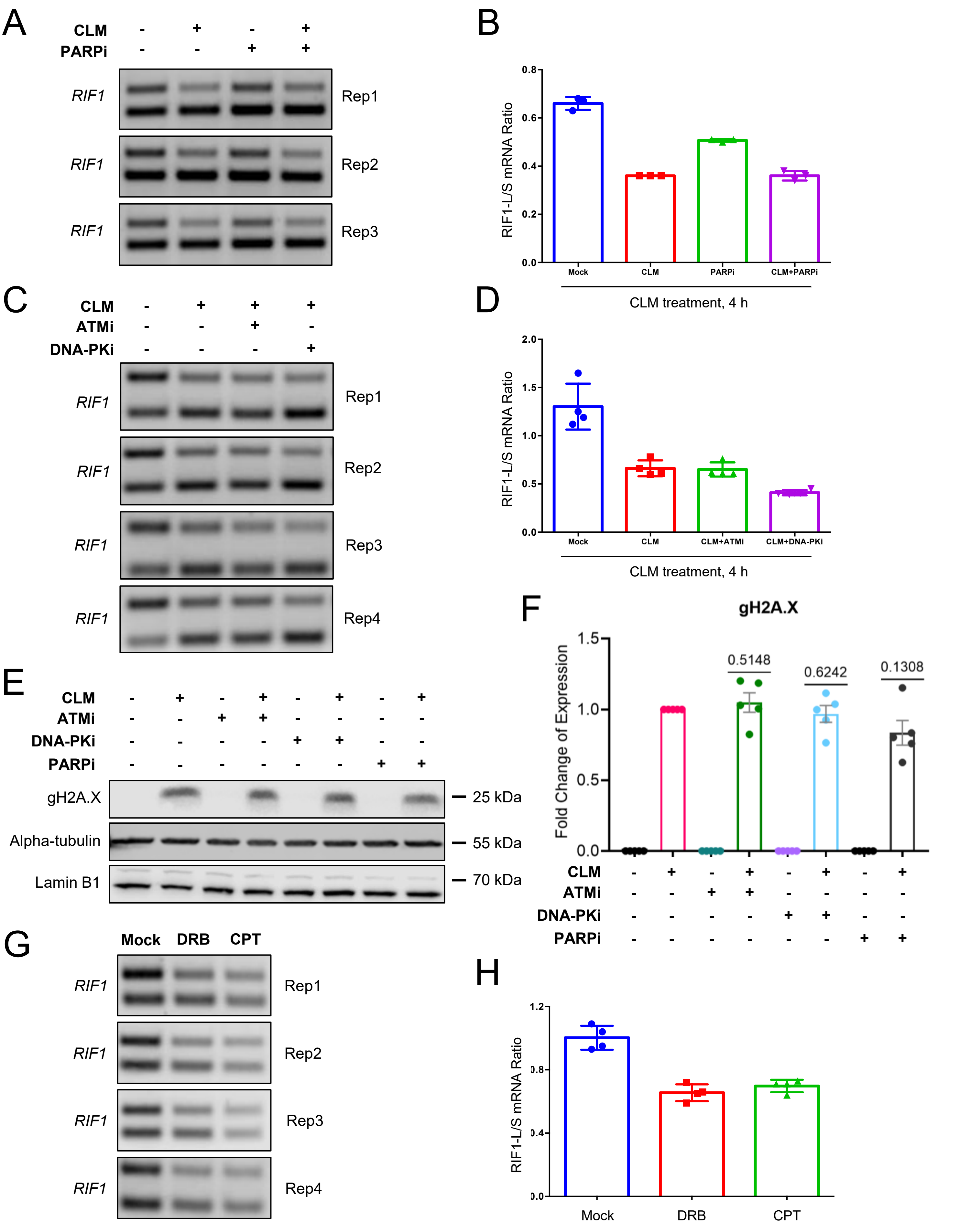
A
CLM - + - +
PARPi - - + +
RIF1
Rep1
Rep2
Rep3
B
RIF1-L/S mRNA Ratio
0.8
0.6
0.4
0.2
0.0
Mock
CLM
PARPi
CLM+PARPi
CLM treatment, 4 h
C
CLM - + + +
ATMi - - + -
DNA-PKi - - - +
RIF1
Rep1
Rep2
Rep3
Rep4
D
RIF1-L/S mRNA Ratio
2.0
1.5
1.0
0.5
0.0
Mock
CLM
CLM+ATMi
CLM+DNA-PKi
CLM treatment, 4 h
E
CLM - + - + - + - +
ATMi - - + + - - - -
DNA-PKi - - - - + + - -
PARPi - - - - - - + +
gH2A.X
Alpha-tubulin
Lamin B1
25 kDa
55 kDa
70 kDa
F
gH2A.X
Fold Change of Expression
1.5
1.0
0.5
0.0
0.5148
0.6242
0.1308
CLM - + - + - + - +
ATMi - - + + - - - -
DNA-PKi - - - - + + - -
PARPi - - - - - - + +
G
Mock DRB CPT
RIF1
Rep1
Rep2
Rep3
Rep4
H
RIF1-L/S mRNA Ratio
1.2
0.8
0.4
0.0
Mock
DRB
CPT

I

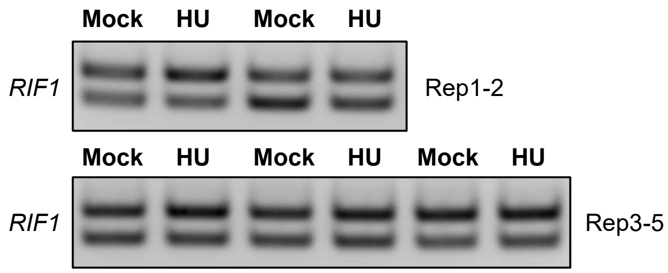
Mock
HU
Mock
HU
RIF1
Rep1-2
Mock
HU
Mock
HU
Mock
HU
RIF1
Rep3-5

J

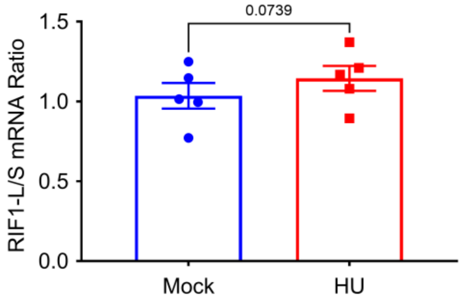
0.0739
RIF1-L/S mRNA Ratio
1.5
1.0
0.5
0.0
Mock
HU

Sup. Fig. 1. Effects of canonical DNA repair inhibitors and replication stress on CLM-dependent *RIF1* alternative splicing. *A*, HeLa cells treated with 10 nM CLM for 4 h with or without pre-treatment of PARP inhibitor (10 μM PJ-34, 1 h) were analyzed by *RIF1* splicing assay. *B*, quantification of the mean RIF1-L/RIF1-S mRNA ratio ± standard error by densitometry from (A). Each *dot* represents an individual biological replicate, N = 3. *C*, HeLa cells treated with 10 nM CLM for 4 h with pre-treatment of either ATM (10 μM KU-55933) or DNA-PK inhibitors (2.5 μM NU7741) for an hour were analyzed by *RIF1* splicing assay. *D*, quantification of the mean RIF1-L/RIF1-S mRNA ratio ± standard error by densitometry from (C). Each *dot* represents an individual biological replicate, N = 4. *E*, representative Western blot image showing the expression level of phospho-histone H2A.X (γH2A.X) in HeLa cells treated with 10 nM CLM with or without the pre-treatment of canonical DNA repair inhibitors as listed in (A,C). Alpha-tubulin and lamin B1 were included as loading controls. *F*, quantification of the mean fold change in γH2A.X expression level ± standard error based on densitometry from (E) in relative to CLM-treated cells. Each *dot* represents an individual biological replicate, N = 5. The *p*-values from two-tailed one sample *t*-test were listed. *G*, Hela cells were treated with either 50 μM 5,6-dichloro-1-beta-D-ribofuranosylbenzimidazole (DRB) or 10 μM Camptothecin (CPT) for 4 h to assess effects of replication stress on *RIF1* splicing. *H*, quantification of the mean RIF1-L/RIF1-S mRNA ratio ± standard error by densitometry from (G). Each *dot* represents an individual biological replicate, N = 4. *I*, HeLa cells were treated with 2 mM of hydroxyurea (HU) for 16 hours for *RIF1* splicing assay. *J*, quantification of the mean RIF1-L/RIF1-S mRNA ratio ± standard error by densitometry from (I). Each *dot* represents an individual biological replicate, N = 5. The *p*-value from paired *t*-test was shown.

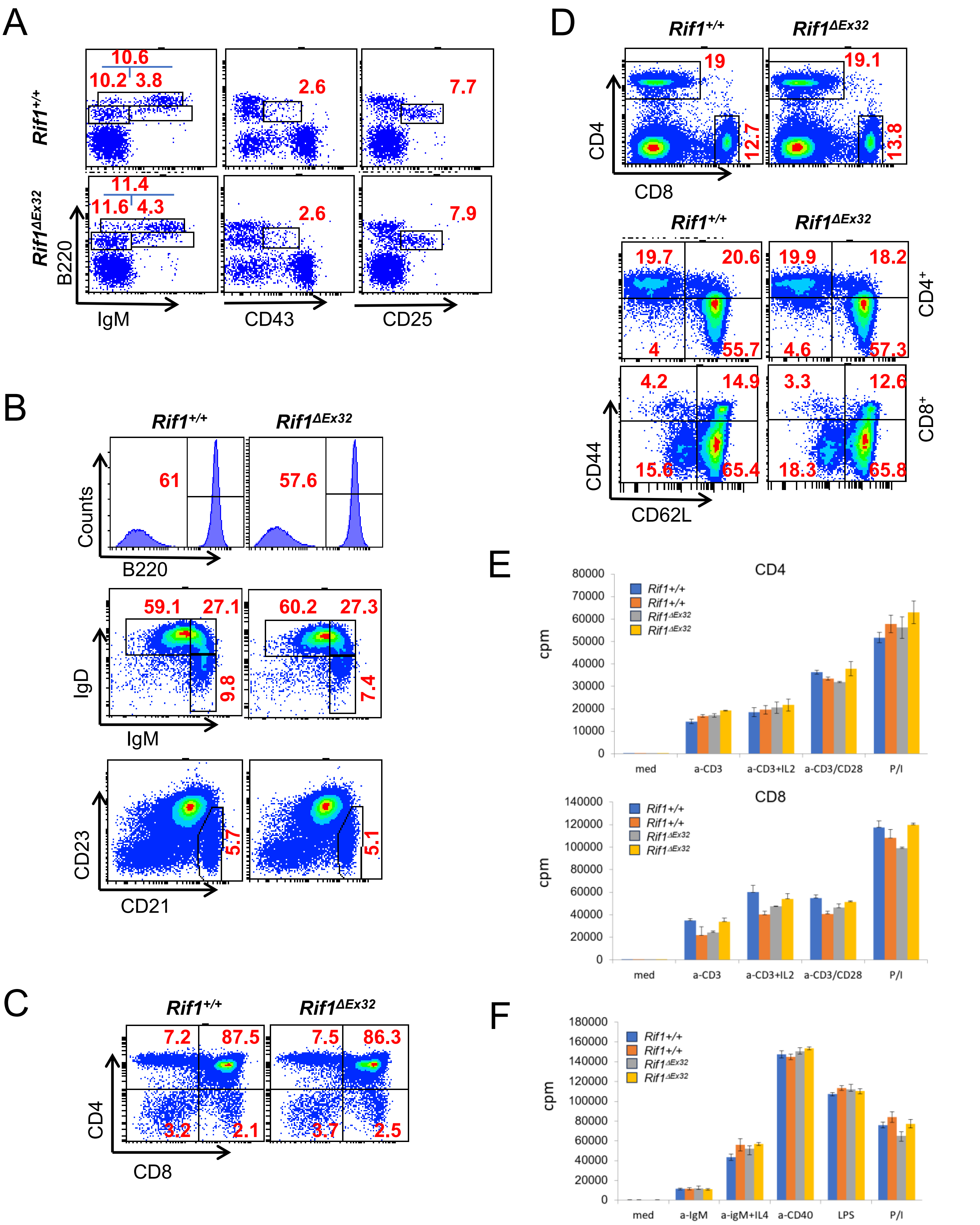
A
Rif1+/+
Rif1ΔEx32
10.6
10.2 3.8
2.6
7.7
11.4
11.6 4.3
2.6
7.9
B220
IgM
CD43
CD25
B
Rif1+/+
Rif1ΔEx32
61
57.6
Counts
B220
59.1 27.1
60.2 27.3
9.8
7.4
IgD
IgM
5.7
5.1
CD23
CD21
C
Rif1+/+
Rif1ΔEx32
7.2 87.5
3.2 2.1
7.5 86.3
3.7 2.5
CD4
CD8
D
Rif1+/+
Rif1ΔEx32
19
12.7
19.1
13.8
CD4
CD8
19.7 20.6
4 55.7
19.9 18.2
4.6 57.3
CD4+
4.2 14.9
15.6 65.4
3.3 12.6
18.3 65.8
CD8+
CD44
CD62L
E
CD4
cpm
Rif1+/+
Rif1+/+
Rif1ΔEx32
Rif1ΔEx32
med
a-CD3
a-CD3+IL2
a-CD3/CD28
P/I
CD8
F
a-IgM
a-igM+IL4
a-CD40
LPS

Sup. Fig. 2. Comparison of T and B cell development in *Rif1*$^{+/+}$ and *Rif1*$^{\Delta Ex32}$ mice. *A*, B cell development in the bone marrow (BM) of *Rif1*$^{+/+}$ and *Rif1*$^{\Delta Ex32}$ mice. BM cells from the mice were stained with anti-B220 and anti-IgM, anti-CD43 or anti-CD25 antibodies. The percentages of B220^{+}IgM^{-} pro/pre-, B220^{+}IgM^{+} immature and B220^{hi}IgM^{+} mature B cells (*left panel*), B220^{+}CD43^{+} pro-B cells (*center panel*), and B220^{+}CD25^{+} pre-B cells (*right panel*) in the gated live cells were shown. *B*, B cell maturation in the spleen of *Rif1*$^{+/+}$ and *Rif1*$^{\Delta Ex32}$ mice. Spleen cells from the mice were stained with anti-B220, anti-IgM, and anti-IgD or anti-B220, anti-CD21, and anti-CD23 antibodies. The percentages of B220^{+} cells in the gated live cells (*top panel*); transitional 1 (T1) (IgM^{hi}IgD^{lo}), transitional 2 (T2) (IgM^{hi}IgD^{hi}), follicular (FO) mature (IgM^{lo}IgD^{hi}) B cells (*center panel*) and marginal zone (MZ) (CD21^{hi}CD23^{lo}) B cells within gated B cell populations (*bottom panel*) were indicated. *C*, T cell development in the thymus of *Rif1*$^{+/+}$ and *Rif1*$^{\Delta Ex32}$ mice. Thymocytes from the mice were stained with anti-CD4 and anti-CD8. The percentages of DN, DP, CD4, and CD8 T cells in the gated live cells were shown. *D*, T cell subpopulations in the spleen of *Rif1*$^{+/+}$ and *Rif1*$^{\Delta Ex32}$ mice. Splenocytes from the mice were stained with anti-CD4, anti-CD8, anti-CD62L and CD44. The percentages of CD4 and CD8 T cells in the gated live cells (*top panel*) and CD62L^{hi}CD44^{lo} naïve, CD62L^{lo}CD44^{hi} effect memory, and CD62L^{hi}CD44^{hi} central memory T cells in the gated CD4^{+} or CD8^{+} cells (*bottom panel*) were shown. *E*, TCR-induced thymidine incorporation in *Rif1*$^{+/+}$ and *Rif1*$^{\Delta Ex32}$ T cells. Splenic CD4 and CD8 T cells sorted from the mice were stimulated with medium (med), anti-CD3, anti-CD3 plus IL-2, anti-CD3 plus anti-CD28, or PMA plus Ionomycin. Proliferative responses were determined by [^{3}H]thymidine incorporation. *F*, BCR-induced thymidine incorporation in *Rif1*$^{+/+}$ and *Rif1*$^{\Delta Ex32}$ B cells. Splenic B cells sorted from the mice were stimulated with medium (med), anti-IgM, or anti-IgM plus IL-4, anti-CD40, LPS, or PMA plus Ionomycin. Proliferative responses were determined by [^{3}H]thymidine incorporation. The data were obtained from five *Rif1*$^{+/+}$ and four *Rif1*$^{\Delta Ex32}$ mice.

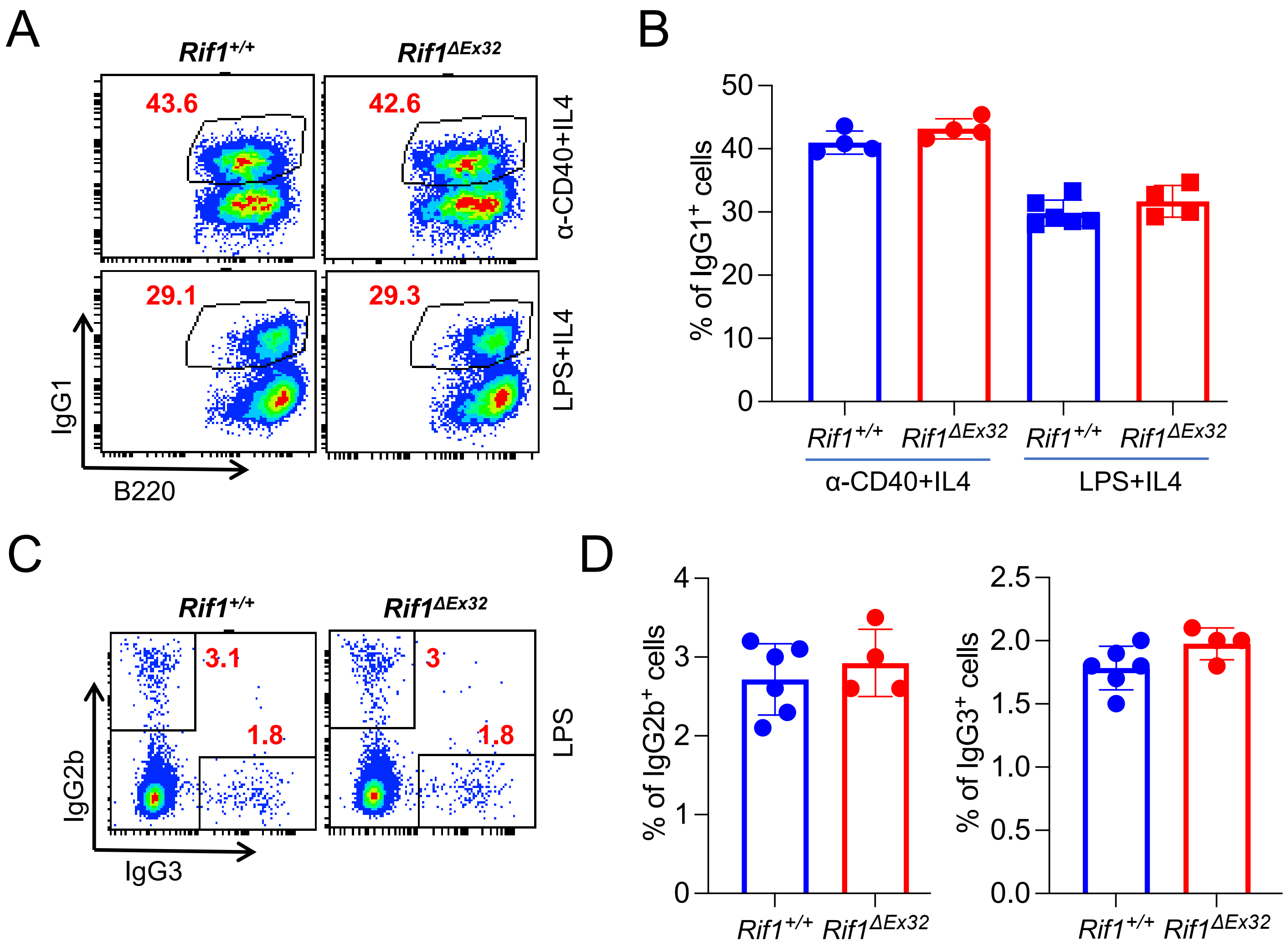
A
Rif1+/+
Rif1ΔEx32
43.6
42.6
29.1
29.3
α-CD40+IL4
LPS+IL4
IgG1
B220
B
% of IgG1+ cells
50
40
30
20
10
0
Rif1+/+
Rif1ΔEx32
Rif1+/+
Rif1ΔEx32
α-CD40+IL4
LPS+IL4
C
Rif1+/+
Rif1ΔEx32
3.1
1.8
3
1.8
LPS
IgG2b
IgG3
D
% of IgG2b+ cells
4
3
2
1
0
Rif1+/+
Rif1ΔEx32
% of IgG3+ cells
2.5
2.0
1.5
1.0
0.5
0.0
Rif1+/+
Rif1ΔEx32

Sup. Fig. 3. Comparison of IgG class switch recombination potential of *Rif1*$^{+/+}$ and *Rif1*$^{\Delta Ex32}$ mice. *A*, *B,* splenic B cells from *Rif1*$^{+/+}$ and *Rif1*$^{\Delta Ex32}$ mice stimulated for four days with CD40 plus IL-4 or LPS plus IL-4 were analyzed for *in vitro* class switch recombination by flow cytometry for IgG1. Percentages of cells in the gated B220^{+} population were shown. *B*, graphical representation of the percentages of IgG1^{+} cells from (A) with mean ± standard deviation. The data were obtained from two or three *Rif1*$^{+/+}$ and 2 *Rif1*$^{\Delta Ex32}$ mice. Each mouse was analyzed in duplicates, and each *dot* represents one replicate. *C*, *D*, splenic B cells from *Rif1*$^{+/+}$ and *Rif1*$^{\Delta Ex32}$ mice stimulated for four days with LPS were analyzed for *in vitro* class switch recombination by flow cytometry for IgG2b and IgG3. Percentages of cells in the gated IgG2b^{+} or IgG3^{+} population were shown. *D*, graphical representation of the percentages of IgG2b^{+} or IgG3^{+} cells from (C) with mean ± standard deviation. Each experiment includes three *Rif1*$^{+/+}$ and two *Rif1*$^{\Delta Ex32}$ mice. Each mouse was analyzed in duplicates, and each *dot* represents one replicate.

A

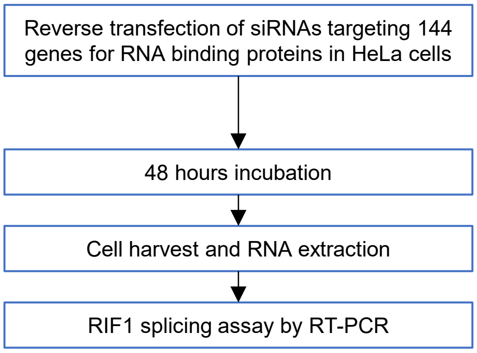

B

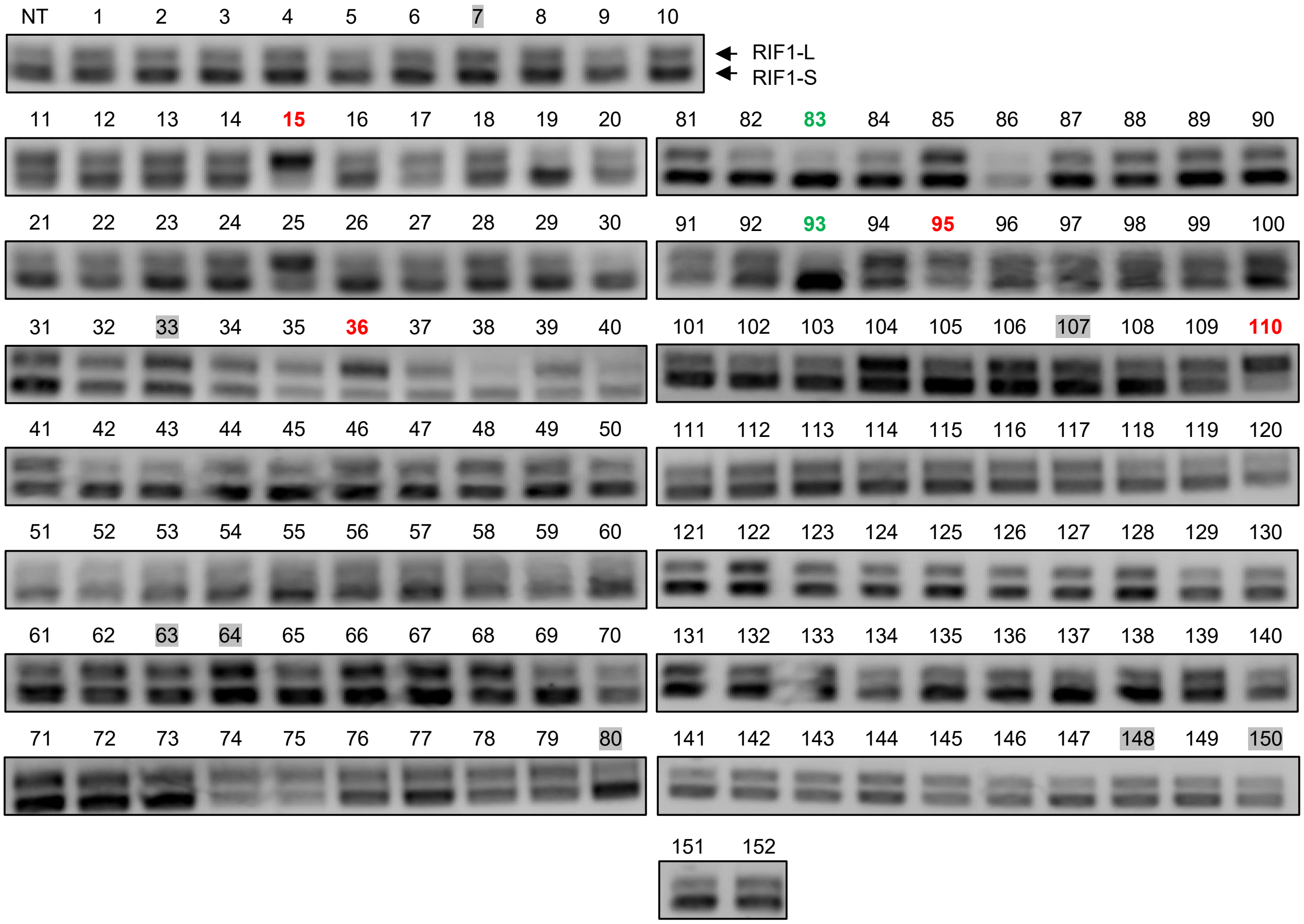

C

Sample ID	Target	RIF1-L/S ratio
NT	Non-targeting control	0.54
15	PTBP1	2.61
36	RBM28	1.33
83	SRSF1	0.16
93	snRNP70	0.25
95	SRSF7	1.5
110	SRSF3	2.83

Sup. Fig. 4. **RNAi screen for *RIF1* splicing regulators.** *A*, schematic of RNAi screen in HeLa cells with siRNAs targeting 144 genes for RNA binding proteins and a non-targeting (NT) siRNA control by reverse transfection. *B*, *RIF1* splicing assay was performed on HeLa cells transfected with the indicated siRNAs (see *Sup. Table 1* for the numbering key used for lane numbering). Lanes highlighted in *grey* were not transfected. *Green labels* denote putative splicing enhancers of Ex32 inclusion; *red labels* denote putative inhibitors of Ex32 inclusion. *C*, candidate *RIF1* splicing regulators chosen for secondary screening by shRNA knockdown based on the changes in RIF1-L/RIF1-S mRNA ratio quantified by densitometry from (B).

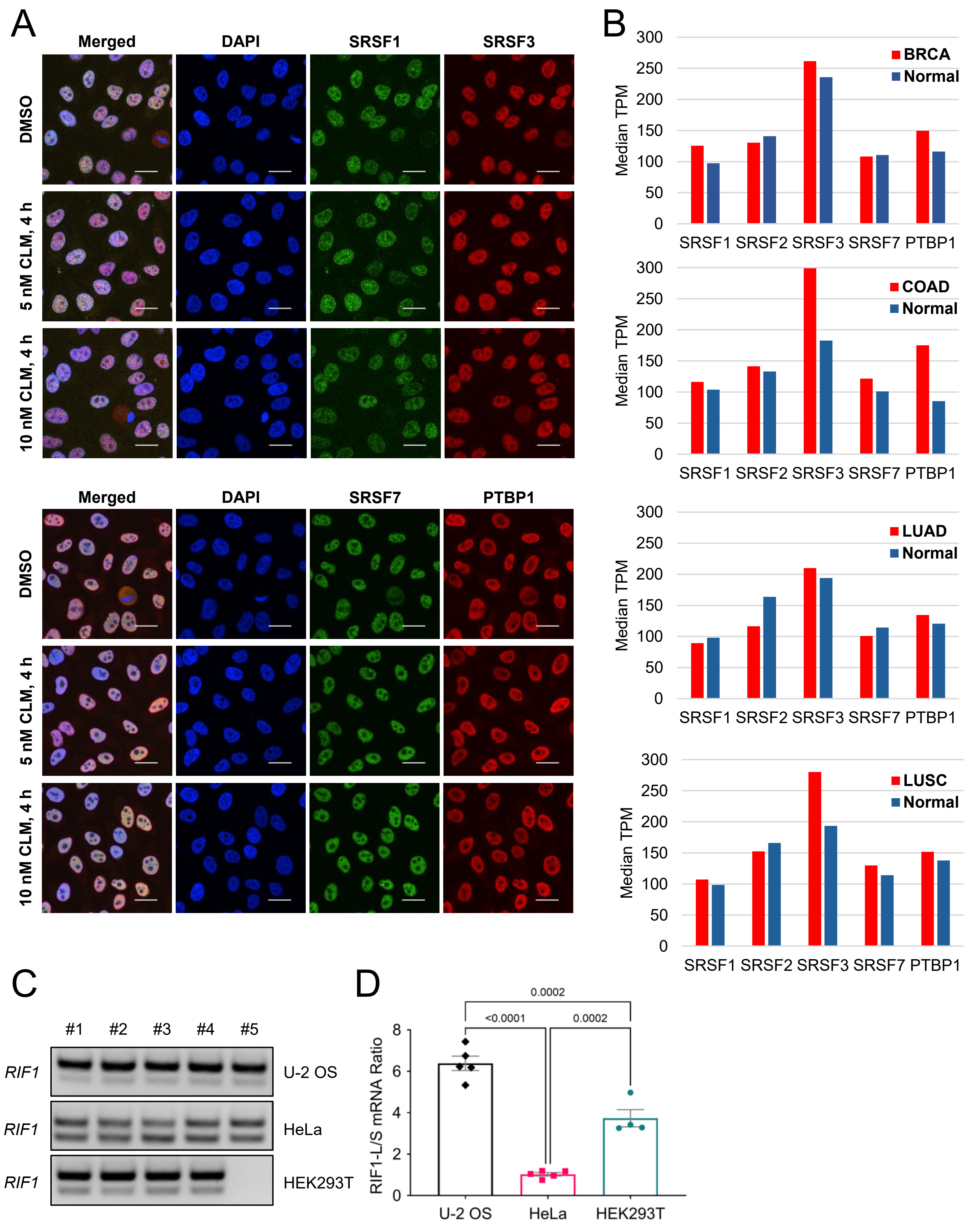
A
Merged
DAPI
SRSF1
SRSF3
DMSO
5 nM CLM, 4 h
10 nM CLM, 4 h
Merged
DAPI
SRSF7
PTBP1
DMSO
5 nM CLM, 4 h
10 nM CLM, 4 h
B
Median TPM
300
250
200
150
100
50
0
BRCA
Normal
COAD
Normal
LUAD
Normal
LUSC
Normal
SRSF1 SRSF2 SRSF3 SRSF7 PTBP1
C
#1 #2 #3 #4 #5
RIF1
U-2 OS
HeLa
HEK293T
D
RIF1-L/S mRNA Ratio
0
2
4
6
8
0.0002
<0.0001
0.0002
U-2 OS
HeLa
HEK293T

E

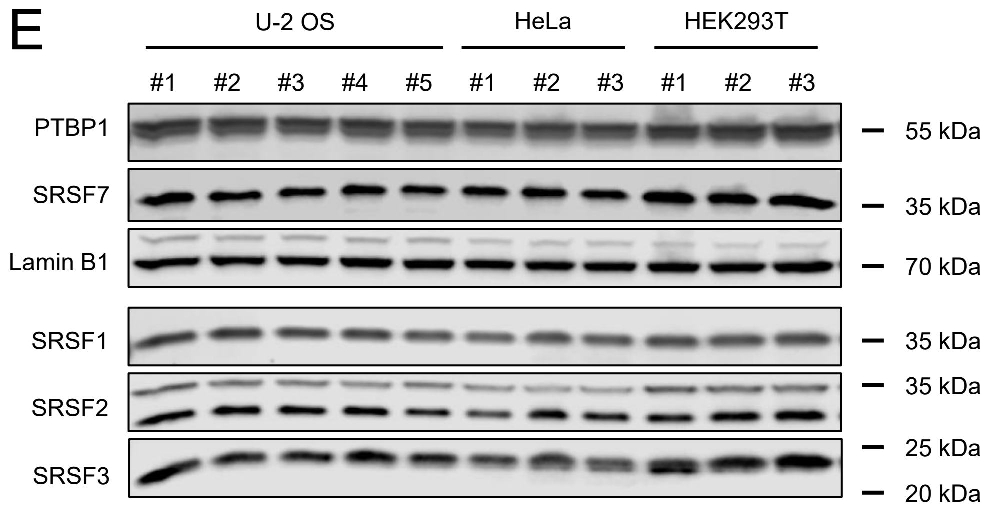
U-2 OS
HeLa
HEK293T
#1 #2 #3 #4 #5 #1 #2 #3 #1 #2 #3
PTBP1
SRSF7
Lamin B1
SRSF1
SRSF2
SRSF3
55 kDa
35 kDa
70 kDa
35 kDa
35 kDa
25 kDa
20 kDa

F

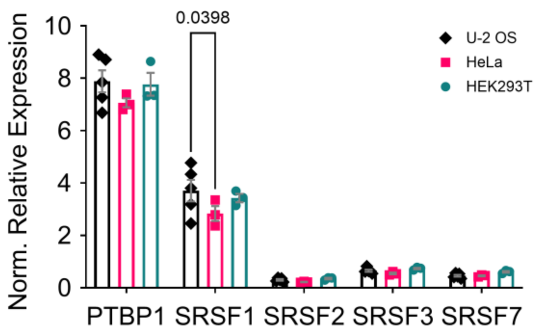
Norm. Relative Expression
10
8
6
4
2
0
0.0398
U-2 OS
HeLa
HEK293T
PTBP1 SRSF1 SRSF2 SRSF3 SRSF7

Sup. Fig. 5. Subcellular colocalization of *RIF1* splicing regulators in response to DNA damage and the expression level in primary cancers and cell lines. *A*, representative images of HeLa cells treated with DMSO, 5 nM or 10 nM of CLM for 4 h before cell fixation and immunofluorescence staining with α-SRSF1 (Abcam ab38017, 1:250), α-SRSF3 (Santa Cruz sc13510, 1:100), α-SRSF7 (Bethyl Laboratories A303-772A, 1:1000), and α-PTBP1 (Invitrogen 32-4800, 1:250). Scale bar = 25 µm. *B*, median expression level in transcript per million (TPM) for *RIF1* splicing regulators – SRSF1, SRSF2, SRSF3, SRSF7, and PTBP1 in four TCGA cancer types of interest (see *Fig. 2* for detailed information of each dataset). Graphs replotted from expression data assessed through GEPIA. *C*, *D*, *RIF1* splicing assay was performed on different cell lines (U-2 OS, HeLa, and HEK293T). Each *dot* represents an individual biological replicate, $4 \leq N \leq 5$. The *p*-values from one-way ANOVA with Tukey's multiple comparisons test was shown. *E*, *F*, The relative expression of *RIF1* splicing regulators (PTBP1, SRSF1, SRSF2, SRSF3, and SRSF7) from the protein samples of (C) were quantified from Western blot images based on densitometry. Each *dot* represents an individual biological replicate, N = 5 for U-2 OS; N = 3 for HeLa and HEK293T. Two-way ANOVA with Tukey's multiple comparisons test was performed, significant *p*-value was listed.

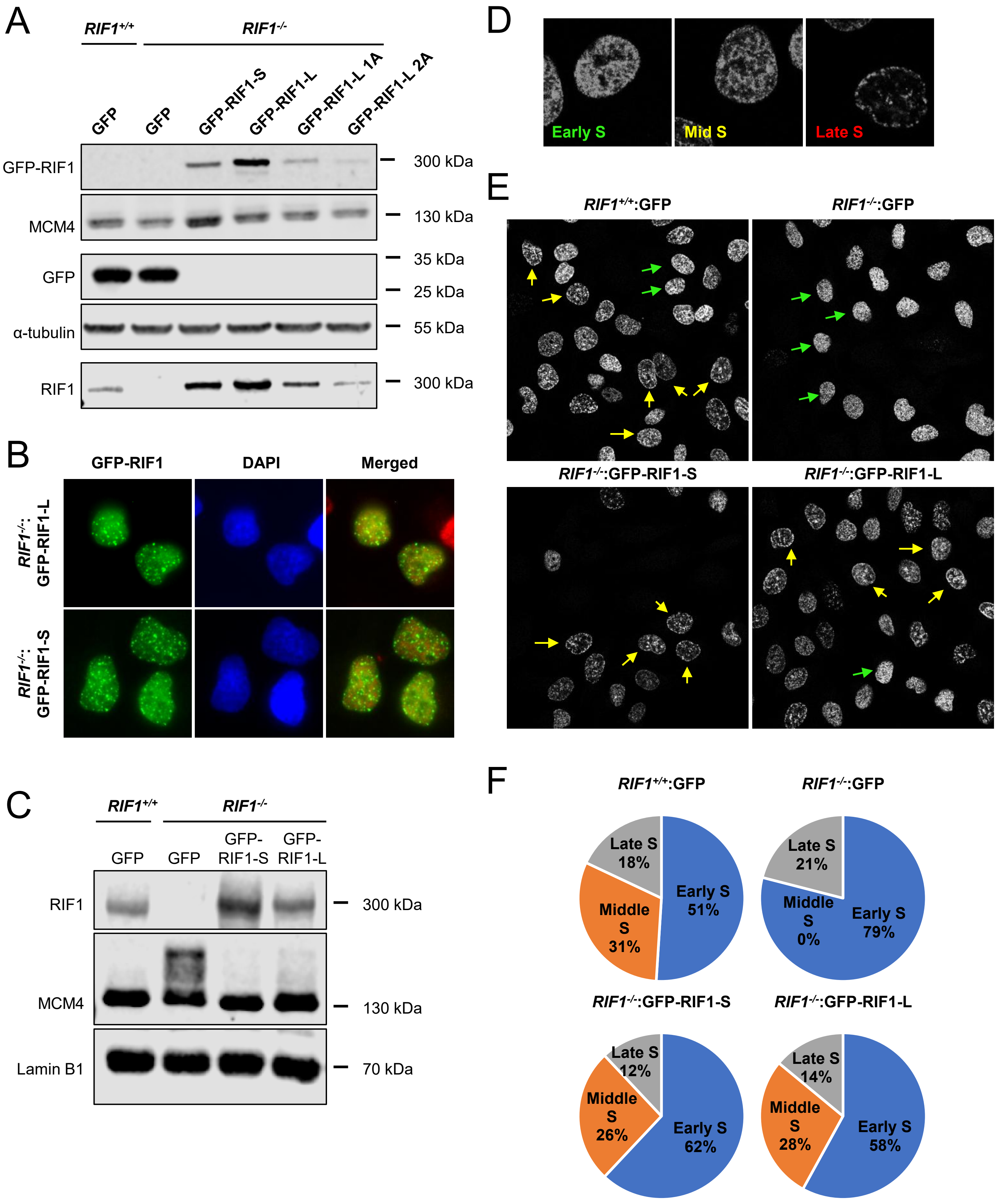
A
RIF1+/+
RIF1-/-
GFP
GFP
GFP-RIF1-S
GFP-RIF1-L
GFP-RIF1-L 1A
GFP-RIF1-L 2A
GFP-RIF1
MCM4
GFP
α-tubulin
RIF1
300 kDa
130 kDa
35 kDa
25 kDa
55 kDa
300 kDa
B
GFP-RIF1
DAPI
Merged
RIF1-/-: GFP-RIF1-L
RIF1-/-: GFP-RIF1-S
C
RIF1+/+
RIF1-/-
GFP
GFP
GFP-RIF1-S
GFP-RIF1-L
RIF1
MCM4
Lamin B1
300 kDa
130 kDa
70 kDa
D
Early S
Mid S
Late S
E
RIF1+/+:GFP
RIF1-/-:GFP
RIF1-/-:GFP-RIF1-S
RIF1-/-:GFP-RIF1-L
F
RIF1+/+:GFP
Late S 18%
Middle S 31%
Early S 51%
RIF1-/-:GFP
Late S 21%
Middle S 0%
Early S 79%
RIF1-/-:GFP-RIF1-S
Late S 12%
Middle S 26%
Early S 62%
RIF1-/-:GFP-RIF1-L
Late S 14%
Middle S 28%
Early S 58%

G

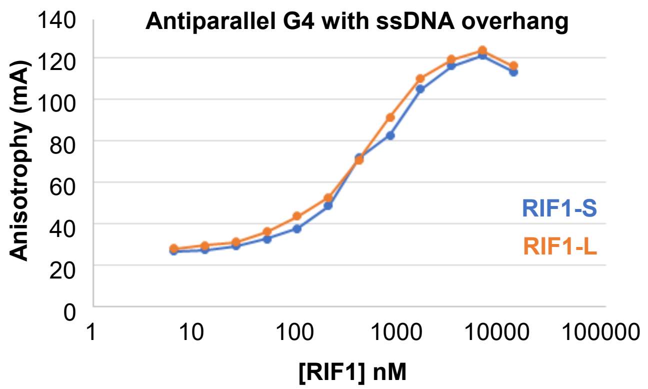
Antiparallel G4 with ssDNA overhang
Anisotrophy (mA)
140
120
100
80
60
40
20
0
1
10
100
1000
10000
100000
[RIF1] nM
RIF1-S
RIF1-L

H

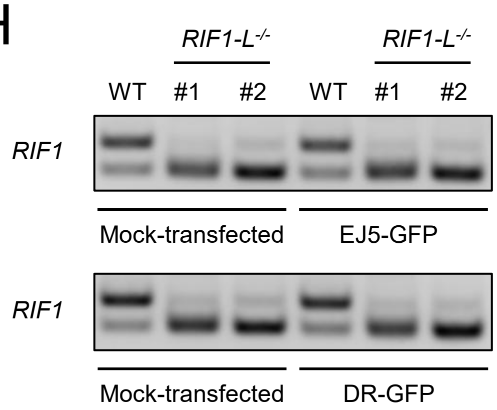
RIF1-L-/-
RIF1-L-/-
WT
#1
#2
WT
#1
#2
RIF1
Mock-transfected
EJ5-GFP
RIF1
Mock-transfected
DR-GFP

I

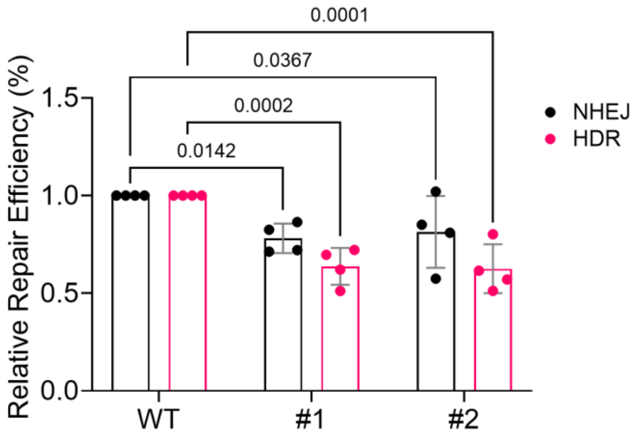
Relative Repair Efficiency (%)
0.0001
0.0367
0.0002
0.0142
1.5
1.0
0.5
0.0
WT
#1
#2
NHEJ
HDR

J

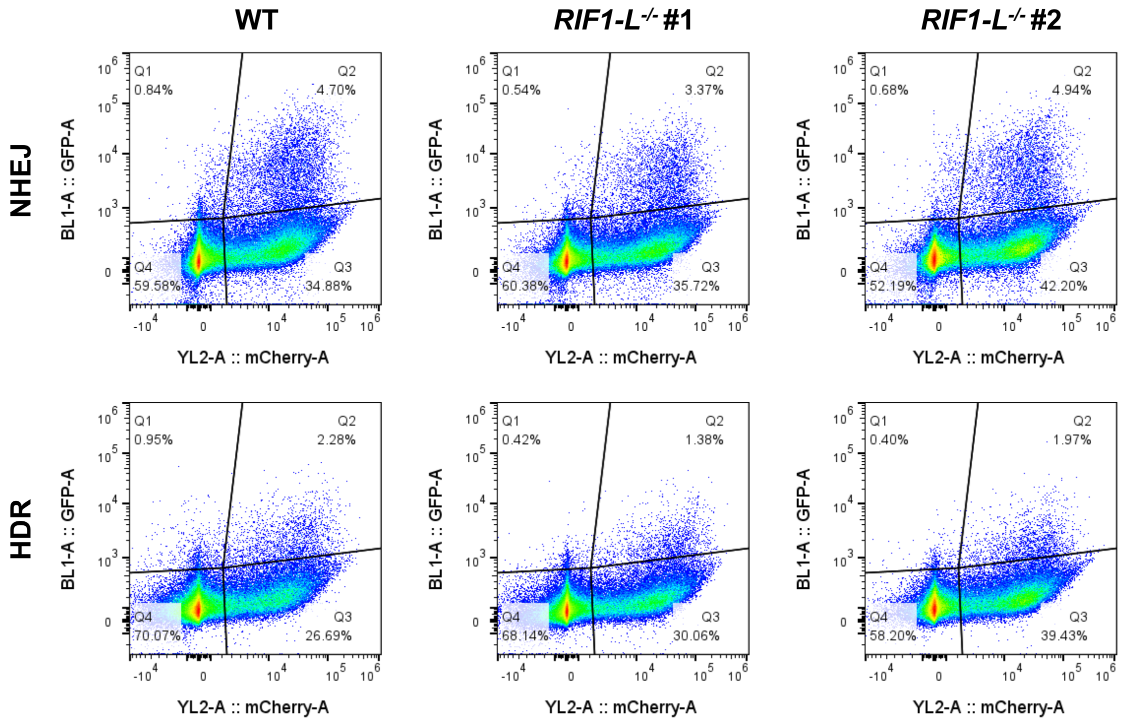
WT
RIF1-L-/- #1
RIF1-L-/- #2
NHEJ
HDR
BL1-A :: GFP-A
YL2-A :: mCherry-A
Q1 0.84%
Q2 4.70%
Q4 59.58%
Q3 34.88%
Q1 0.54%
Q2 3.37%
Q4 60.38%
Q3 35.72%
Q1 0.68%
Q2 4.94%
Q4 52.19%
Q3 42.20%
Q1 0.95%
Q2 2.28%
Q4 70.07%
Q3 26.69%
Q1 0.42%
Q2 1.38%
Q4 68.14%
Q3 30.06%
Q1 0.40%
Q2 1.97%
Q4 58.20%
Q3 39.43%

Sup. Fig. 6. RIF1-L and RIF1-S isoforms have similar activity in canonical measures of RIF1 function. *A*, Western blot analysis of *RIF1*$^{+/+}$ U-2 OS cells and *RIF1*$^{-/-}$ U-2 OS cells stably transfected with plasmid vectors encoding GFP, GFP-RIF1-L, GFP-RIF1-S, GFP-RIF1-L^{1A} and RIF1-L^{2A} alleles. Both α-GFP (Santa Cruz sc9996, 1:200) and α-RIF1 (Bethyl Laboratories A300-569A; 1:500) detected a band of ~300 kDa for GFP-RIF1 alleles. MCM4 and α-tubulin were included as loading controls. *B*, RIF1-L and RIF1-S are recruited to IR-induced foci with comparable efficiency. *RIF1*$^{-/-}$:GFP-RIF1-S and *RIF1*$^{-/-}$:GFP-RIF1-L U-2 OS cells were exposed to 10 Gy IR and stained with DAPI three hours post-irradiation prior to imaging. *C*, MCM4 (Santa Cruz sc28317, 1:100 dilution) hyperphosphorylation in *RIF1*$^{-/-}$ U-2 OS cells were rescued by stable expression of either GFP-RIF1-S or GFP-RIF1-L. Lamin B1 was included as loading control. *D*, *E*, RIF1-L and RIF1-S rescued the DNA replication pattern defect of *RIF1*$^{-/-}$ U-2 OS cells. Asynchronous U-2 OS cells of the indicated genotypes in (E) were pulse-labeled with EdU for 20 min and scored for the presence of early, mid, or late EdU staining patterns, as depicted in *panel* (D). *E*, representative EdU incorporation patterns of *RIF1*$^{-/-}$:GFP-RIF1-S and *RIF1*$^{-/-}$:GFP-RIF1-L U-2 OS cells. Cells exhibiting early and mid S-phase EdU incorporation patterns are denoted by *green* and *yellow arrows* respectively. *F*, quantification of the percentage of cells in early, mid, and late S-phase patterns as shown in (D) using a minimum of 100 cells per genotype. Note the lack of mid S-phase replication patterns in *RIF1*$^{-/-}$ cells that were rescued by both RIF1-L and RIF1-S. *G*, RIF1^{CTD}-S and RIF1^{CTD}-L binds to antiparallel G4 quadruplex substrate with equal anisotropy *in vitro*. *H*, *RIF1* splicing assay of WT HEK293T and its CRISPR/Cas9-generated *RIF1-L*$^{-/-}$ clones (#1 and #2) sampled from the cells used for the reporter assays. *I*, cells were mock transfected with I-Sce I-expressing plasmid or transfected with either EJ5-GFP or DR-GFP, mCherry, and I-Sce I-expressing plasmids. After 72 hours, the percentage of GFP+ and mCherry+ cells were analyzed by flow cytometer. The repair efficiency was estimated by the total percentage of GFP+ cells (GFP+ mCherry- and GFP+ mCherry+) normalized to the total percentage of mCherry+ cells (GFP- mCherry+ and GFP+ mCherry+) to account for transfection efficiency. The mean fold change in repair efficiency ± standard error of clone #1 and #2 was calculated in relative to WT, N = 4. Repeated measures two-way ANOVA and Dunnett's multiple comparisons test was performed and the resulting *p*-values were listed. *J*, representative images showing the percentages of GFP and mCherry positive cells in WT, *RIF1-L*$^{-/-}$ clones #1 and #2 in the NHEJ (EJ5-GFP) and HDR (DR-GRP) reporter assays.

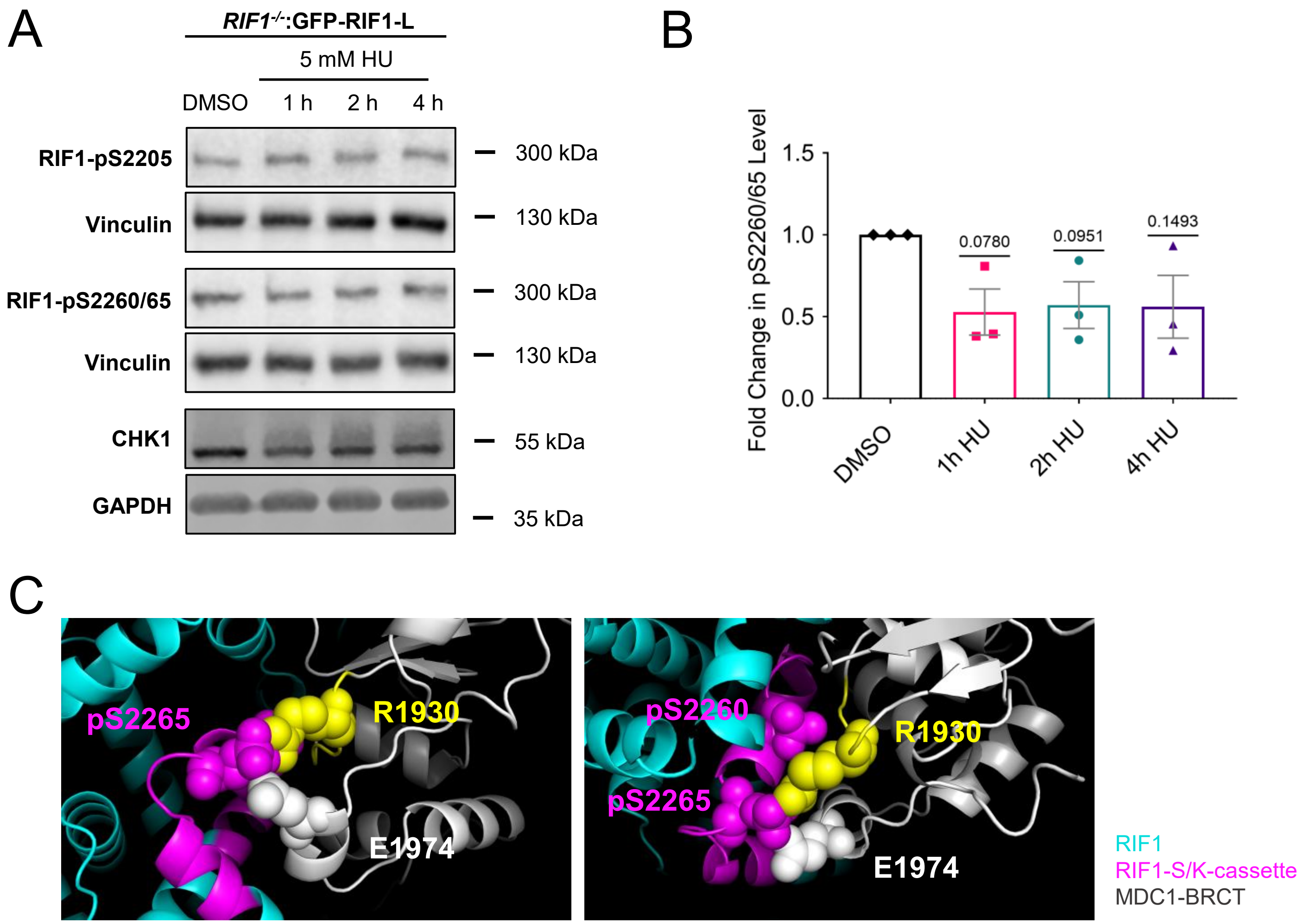
A
RIF1-/-:GFP-RIF1-L
5 mM HU
DMSO
1 h
2 h
4 h
RIF1-pS2205
Vinculin
RIF1-pS2260/65
Vinculin
CHK1
GAPDH
300 kDa
130 kDa
300 kDa
130 kDa
55 kDa
35 kDa
B
Fold Change in pS2260/65 Level
1.5
1.0
0.5
0.0
0.0780
0.0951
0.1493
DMSO
1h HU
2h HU
4h HU
C
pS2265
R1930
E1974
pS2260
R1930
pS2265
E1974
RIF1
RIF1-S/K-cassette
MDC1-BRCT

Sup. Fig. 7. RIF1 phosphorylations are not induced by hydroxyurea but predicted to interact with the MDC1-BRCT domain. *A*, *RIF1*$^{-/-}$:GFP-RIF1-L U-2 OS cells were treated with 5 mM hydroxyurea (HU) for the indicated timepoint prior to immunoblotting analysis of RIF1-pS2205 and RIF1-pS2260/65 phosphorylation level. CHK1 was included as experimental control. Vinculin and GAPDH were included as loading controls. *B*, quantification of RIF1-pS2260/65 level from (A) based on densitometry. *Bar height* represents mean fold change in RIF1-pS2260/65 relative to the baseline phosphorylation level in DMSO control ± standard error. Each *dot* represents an individual biological replicate, N = 3. The *p*-values from two-tailed one sample *t*-test were listed. *C*, RIF1-L protein sequence phosphorylated on the seven CDK1 phosphosites (*cyan ribbon*) and MDC1 protein sequence (*grey ribbon*) were used as the inputs for AlphaFold 3 structure prediction ran on Google DeepMind's AlphaFold Server on 2025-01-17. Five iterations of modelling were performed, and the last output model was visualized in PyMOL v3.1.1. RIF1-pS2260/65 was shown in *magenta*, MDC1-R1930 in *yellow*, and MDC1-E1974 in *grey filled spheres*.

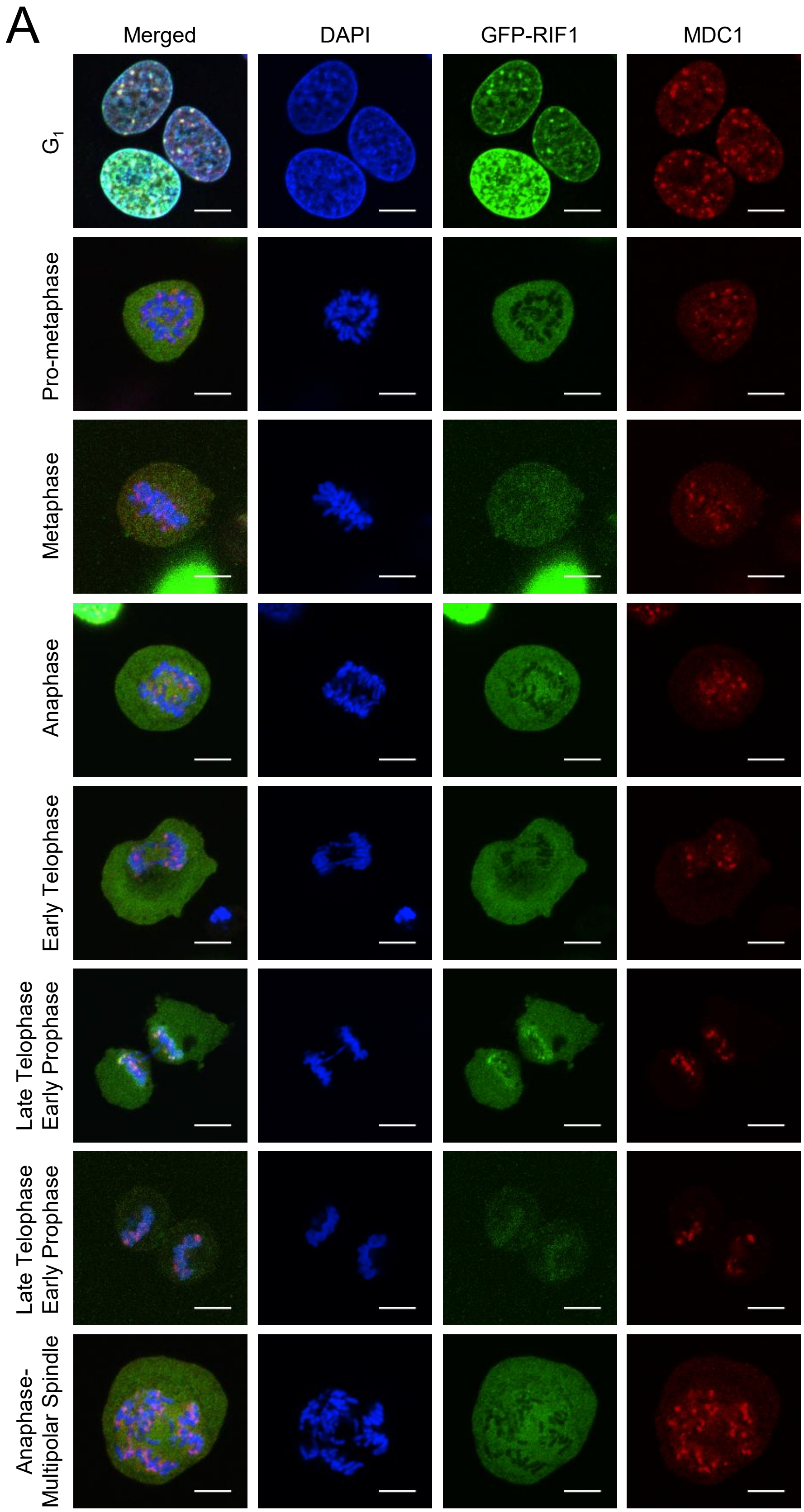
A
Merged
DAPI
GFP-RIF1
MDC1
G_1
Pro-metaphase
Metaphase
Anaphase
Early Telophase
Late Telophase
Early Prophase
Late Telophase
Early Prophase
Anaphase-
Multipolar Spindle

Sup. Fig. 8. RIF1 is evicted from mitotic chromosomes. *A*, *RIF1*$^{-/-}$:GFP-RIF1-L U-2 OS cells were treated with 100 ng/ml Nocodazole for 16 hours followed by 1 Gy irradiation and 1 h recovery at 37 °C. The cells were stained with MDC1 antibody (Sigma HPA006915, 1:500 dilution). Note the eviction of RIF1 from mitotic chromosomes since pro-metaphase and the reassociation of RIF1 on the chromosomes during late telophase/early prophase. Scale bar = 10 μm.
