# Supplementary material for: DNA-damage dependent isoform switching modulates RIF1 DNA repair complex assembly and phase separation
Source: J Biol Chem. 2025 Oct 24;301(12):110857. doi: 10.1016/j.jbc.2025.110857 (PMC12702018; doi:10.1016/j.jbc.2025.110857)
Supplement: Supporting Video legend [file mmc9.pdf]

SUPPLEMENTARY VIDEOS
https://github.com/adenine-koo/RIF1_Raw-Sup_data

Sup. Video 1. Three-dimensional reconstruction of RIF1^{CTD}-S anisosomes which resembled oblong spheroids from 10 x 1 μm image sections taken at ~ 25 s interval for a total of 534 s.

Sup. Video 2. Three-dimensional reconstruction of RIF1^{CTD}-S anisosomes from 7 x 2 μm image sections taken at 10.7 s interval for a total of 311 s, showing the dynamics of anisosomes and fusion events.

Sup. Video 3. RIF1^{CTD}-L^{5KQ} (and other RIF1^{CTD}-L variants) formed nested anisosome structures in which at least one smaller anisosome was formed within a bigger anisosome. Acquisition time interval = 1.1 s, scale bar = 10 μm. Note that this is a video from FRAP experiment so there was a bleaching event of 2 s before Frame #3.

Sup. Video 4. FRAP video images of RIF1^{CTD}-S. *Red arrow* marked the bleached anisosome. Scale bar = 10 μm.

Sup. Video 5. FRAP video images of RIF1^{CTD}-L. *Red arrow* marked the bleached anisosome. Scale bar = 10 μm.
